# Efficacy and safety of PD-1 inhibitors as second-line treatment for advanced squamous esophageal cancer: a systematic review and network meta-analysis with a focus on PD-L1 expression levels

**DOI:** 10.3389/fimmu.2024.1510145

**Published:** 2025-01-23

**Authors:** Fei Yang, Min Dan, Jindan Shi, Ling Fan, Haoluo Zhang, Tiantian Jian, Kelu Lei, Yue Wang, Juan Xin, Zhigang Yu, Wei Chen

**Affiliations:** ^1^ Department of Pharmacy, Ya ‘an People’s Hospital, Ya ‘an, China; ^2^ Department of Pharmacy, Emergency General Hospital, Beijing, China

**Keywords:** esophageal squamous cell carcinoma (ESCC), immune checkpoint inhibitors (ICIs), network meta-analysis, efficacy, safety

## Abstract

**Background:**

PD-1 inhibitors have shown promising efficacy in enhancing OS and AEs as second-line therapies for patients with advanced esophageal squamous cell carcinoma (ESCC). However, there remains no clear consensus on which PD-1 inhibitor provides the best balance between efficacy and safety. To address this key issue in the second-line treatment of ESCC, we conducted a network meta-analysis (NMA) with a focus on OS benefits, particularly in patients with different levels of PD-L1 expression.

**Methods:**

A systematic search of relevant literature was conducted in Web of Science, Embase, PubMed, and Cochrane Library, covering publications from the inception of these database to June 2024. The evaluated endpoints included OS, progression-free survival (PFS), objective response rate (ORR), AEs, and Grade ≥ 3 adverse events (Grade ≥ 3 AEs). A systematic review and Bayesian network meta-analysis were performed to assess the efficacy and safety of various immunotherapy regimens in patients with advanced ESCC. To ensure transparency, novelty, and reliability, this study was prospectively registered in the systematic review registry (CRD42024540581).

**Results:**

Five randomized controlled trials (RCTs), encompassing 2,078 patients and six treatment regimens, were included in this study. Among advanced ESCC patients not selected based on PD-L1 expression, Sintilimab demonstrated the greatest OS benefit (HR = 0.70, 95% CI: 0.50-0.98). Camrelizumab showed the most favorable improvement in PFS compared to chemotherapy (HR = 0.64, 95% CI: 0.47-0.87) and also achieved the best ORR benefit (OR = 3.72, 95% CI: 1.98-6.99). In terms of safety, Nivolumab (OR = 0.10, 95% CI: 0.05-0.19) and Tislelizumab (OR = 0.18, 95% CI: 0.10-0.33) exhibited significant safety advantages over chemotherapy concerning AEs. Moreover, Nivolumab (OR = 0.13, 95% CI: 0.08-0.20) was associated with a markedly lower risk of Grade ≥ 3 AEs compared to chemotherapy. Subgroup analysis based on PD-L1 expression revealed that Tislelizumab (HR = 0.53, 95% CI: 0.37-0.76) offered the greatest OS benefit for patients with PD-L1 ≥ 10%, while Camrelizumab (HR = 0.71, 95% CI: 0.57-0.89) was the most likely regimen to provide an OS advantage for patients with PD-L1 < 10%.

**Conclusion:**

Compared to chemotherapy, PD-1 inhibitors may provide improved survival outcomes for patients with advanced ESCC. Among patients not selected based on PD-L1 expression, Sintilimab is most likely to deliver the best survival benefit. For patients with PD-L1 expression ≥ 10%, Tislelizumab is expected to offer the greatest efficacy, while Camrelizumab appears to be the most effective for those with PD-L1 < 10%.

**Systematic Review Registration:**

https://www.crd.york.ac.uk/PROSPERO/, identifier CRD42024540581.

## 1 Introduction

Esophageal cancer is the 11th most prevalent cancer globally and the 7th leading cause of cancer-related deaths (1). Esophageal cancer poses an enormous healthcare burden, with an average of more than 400,000 deaths per year, while at the same time approximately 470,000+ new cases are created each year (2). ESCC is the most common histological subtype, particularly in populations from Asia and East Africa, representing 90% of esophageal cancer cases (3). More than half of global ESCC cases occur in China (4). The asymptomatic nature of ESCC in its early stages often leads to diagnosis at a locally advanced stage. The current 5-year survival rate for patients with locally advanced ESCC is about 10-20% (5). Furthermore, the majority of patients experience early recurrence or metastasis, leading to a poor prognosis for those with advanced ESCC (6–8).

Second-line treatments, including monotherapy with paclitaxel, docetaxel, or irinotecan, are commonly used after the failure of first-line therapies in advanced or metastatic ESCC. As second-line treatment usually implies disease progression in patients, accompanied by a decline in function, worsening of symptoms and quality of life (9), the use of chemotherapy induces DNA damage with the risk of secondary malignancy. At the same time chemotherapy-induced side effects of nausea, vomiting, anemia and increased risk of infections make the efficacy obtained with second-line treatment with chemotherapy unsatisfactory, with overall survival typically less than 10 months (10, 11). Therefore, there is a critical need to explore innovative therapies that can improve the prognosis for patients with advanced ESCC.

Tumor immunotherapy is a treatment modality that controls and kills tumor cells by repairing and enhancing the function of the body’s immune system (12). Since the FDA approval of immune checkpoint inhibitor antibodies targeting CTLA-4 in 2011, there has been a renewed interest in the immune system for the treatment of a wide range of malignancies. Over the next few years, more antibodies targeting immune checkpoint inhibitors such as PD-1, PD-L1, and LAG-3 entered clinical practice (13), offering renewed hope to patients (14). These inhibitors work by attaching to protein receptors on the surface of T cells, which activates T cell-mediated immune responses, blocking the PD-L1/PD-1 inhibitory signaling pathway, and enhancing the body’s anti-tumor immune response (15, 16). The inflammatory microenvironment of squamous esophageal cancer is capable of enriching immunosuppressive T regulatory cells, which makes immunotherapy potentially surprisingly effective in treating squamous esophageal cancer (17). Programmed cell death protein 1 (PD-1) is the most prevalent immune checkpoint in ESCC, and its inhibition by monoclonal antibodies has shown therapeutic efficacy (18). Results from the ATTRACTION-3 (19), KEYNOTE-181 (20), and ESCORT (21) studies demonstrate that PD-1 inhibitors, In second-line treatment regimens, PD-1 inhibitors are associated with longer overall survival and higher overall remission rates compared to standard chemotherapy. Also PD-1 inhibitors have a more manageable safety profile with a lower incidence of treatment-related adverse events (22–24). Currently in network meta-analyses, only cost-effectiveness analyses of PD-1 inhibitors as second-line treatment for advanced squamous esophageal cancer have been performed (25, 26), however, no consensus has been reached on the optimal choice of PD-1 inhibitors for second-line treatment of patients with advanced ESCC in terms of efficacy and safety.

With the increasing use of PD-1 inhibitors in ESCC, most RCTs have directly compared the outcomes of PD-1 inhibitors with chemotherapy (27, 28). As a result, there is an urgent need to develop strategies for optimizing PD-1 inhibitor treatment regimens to inform the design of future head-to-head clinical trials.

This study aims to evaluate the efficacy and safety of all currently available second-line PD-1 inhibitors in patients with advanced ESCC. Utilizing a Bayesian framework for comparisons of the effects of all second-line PD-1 inhibitors on survival in patients with advanced ESCC and to rank the use of these treatments in patients (29). Additionally, a systematic review and meta-analysis will be conducted to identify the optimal treatment strategy based on varying levels of PD-L1 expression.

## 2 Materials and methods

This network meta-analysis (NMA) adheres to the Preferred Reporting Items for Systematic Reviews and Meta-Analyses (PRISMA) extension guidelines for network meta-analyses (**Supplementary Table 1**) (30). In the absence of randomized controlled trials directly comparing different PD-1 inhibitors, the Bayesian methods indirectly compare and predict the probability of efficacy and safety of different PD-1 inhibitors given the probability distributions of the model parameters of the observational data and the reference values of the prior beliefs of the external information (31). The study protocol has been registered on the PROSPERO platform to guarantee transparency, reliability, and innovation (Registration No: CRD42024540581) (32).

### 2.1 Data sources and search strategy

A thorough search of the literature was performed across the Web of Science, Embase, PubMed, and Cochrane Library databases. Search terms included advanced squamous cell esophageal cancer, immune checkpoint inhibitors, PD-1 receptor, PD-1 inhibitors, and randomized controlled trials. To ensure thoroughness, we also reviewed references from relevant published studies and review articles to address any gaps in the keyword search. The search period spanned from the inception of the databases to December 2024, utilizing a combination of free-text and subject heading search strategies. The specifics of the database search strategy can be found in **Supplementary Table 2**.

### 2.2 Selection criteria

#### 2.2.1 Inclusion criteria

1. Randomized controlled trials of locally advanced or metastatic ESCC that are refractory or intolerant to first-line treatment;
2. Treatment groups receiving PD-1 inhibitors must utilize a single PD-1 inhibitor, while the chemotherapy group must use standard second-line chemotherapy agents for ESCC;
3. Trials must report at least three of the following outcomes: OS, PFS, ORR, and the incidence of treatment-related AEs or Grade ≥ 3 AEs as outcome measures. This ensures a comprehensive assessment of the efficacy and safety of PD-1 inhibitors.
4. Correlation data between PD-L1 expression and clinicopathologic parameters are available.
5. Sufficient survival data are available to estimate prognosis, such as minimum follow-up time.

#### 2.2.2 Exclusion criteria

1. Exclude reviews or case reports;
2. Exclude duplicate randomized controlled trials;
3. Exclude non-randomized controlled trials and animal studies;
4. Exclude randomized controlled trials with unclear outcome measures.

All included randomized controlled trials were independently reviewed and verified by two reviewers to ensure the data were up to date. When clinical trial results were published in different journals or in different years, the article with the most complete data was selected. Any discrepancies were resolved through group discussion.

### 2.3 Data extraction

Two investigators (FY and MD) independently extracted data from the RCTs in compliance with PRISMA guidelines, with any disagreements resolved through consensus with a third investigator (WC). The data extracted from each article included trial name, publication source, design, randomization ratio, trial phase, publication year, sample size, clinical trial registration number, treatment regimens for both experimental and control groups, patient age, sex distribution, histological type, PD-L1 expression, patient ethnicity, ECOG status, and disease state. The key outcome measures were summarized, including hazard ratios (HR) with their respective 95% confidence intervals (95% CI) for OS and PFS, along with odds ratios (OR) with 95% CI for ORR, the incidence of any AEs, and Grade ≥ 3 AEs.

### 2.4 Quality assessment

The risk of bias in network meta-analysis (RoB NMA) tool project aims to develop the first tool to assess risk of bias in a review with network meta-analyses (NMAs) (33). This study utilized the Cochrane tool as the primary method to assess both the quality and potential risk of bias. The evaluation focused on five core areas: bias linked to the randomization process, deviations from planned interventions, incomplete reporting of outcomes, biases in outcome measurement, and selective result reporting. Two reviewers independently carried out the assessments, while a third reviewer mediated and resolved any conflicts. After the final review, the randomized controlled trials (RCTs) were grouped into three risk levels: low risk, high risk, and moderate concerns. All included RCT studies had a relatively low risk (34). Two independent authors evaluated the included articles, and another author made the final decision on the controversial parts.

### 2.5 Statistical analysis

The primary outcomes were OS, PFS, and ORR. Secondary outcomes included AEs and Grade ≥ 3 AEs. HR with 95% CI were used to measure effect sizes for OS and PFS, while OR with 95% CI were used for ORR, AEs, and Grade ≥ 3 AEs.

NMA was performed using a Bayesian framework with the ‘rjags’ and ‘gemtc’ packages in R software (35). A fixed-effect model was applied, utilizing three independent Markov chains, each running 10,000 burn-in iterations followed by 30,000 sample iterations. HR and OR were utilized as effect size metrics to rank the efficacy and safety of various treatment regimens, based on the iterations of the Markov chains, with the results displayed visually.

In the absence of direct head-to-head studies among the various PD-1 inhibitor groups, this study employed NMA for indirect comparisons. To ensure the accuracy of the NMA’s indirect comparisons, pairwise meta-analyses based on frequentist methods were conducted for the available head-to-head studies, and the results were compared with the corresponding Bayesian framework summaries (**Supplementary Table 3**).

This study employed RevMan 5.3 software to conduct pairwise meta-analyses using frequentist methods, comparing the efficacy and safety of second-line immunotherapy to standard chemotherapy (36). Heterogeneity was assessed using the Q test and the *I²* statistic, with *I²* ≤ 50% or *P* ≥ 0.1 indicating low heterogeneity and *I²* > 50% or *P* < 0.1 indicating high heterogeneity (37). For studies with low heterogeneity, a fixed-effect model was employed, whereas a random-effects model was utilized for those with high heterogeneity. Sensitivity analyses were also conducted for studies with high heterogeneity by sequentially excluding studies that had a significant impact on heterogeneity. This approach allowed for the comparison of efficacy and safety both before and after exclusions, along with statistical significance assessments. Funnel plots were used to evaluate potential publication bias.

## 3 Results

### 3.1 Systematic review and characteristics of the included studies

**Figure 1:**
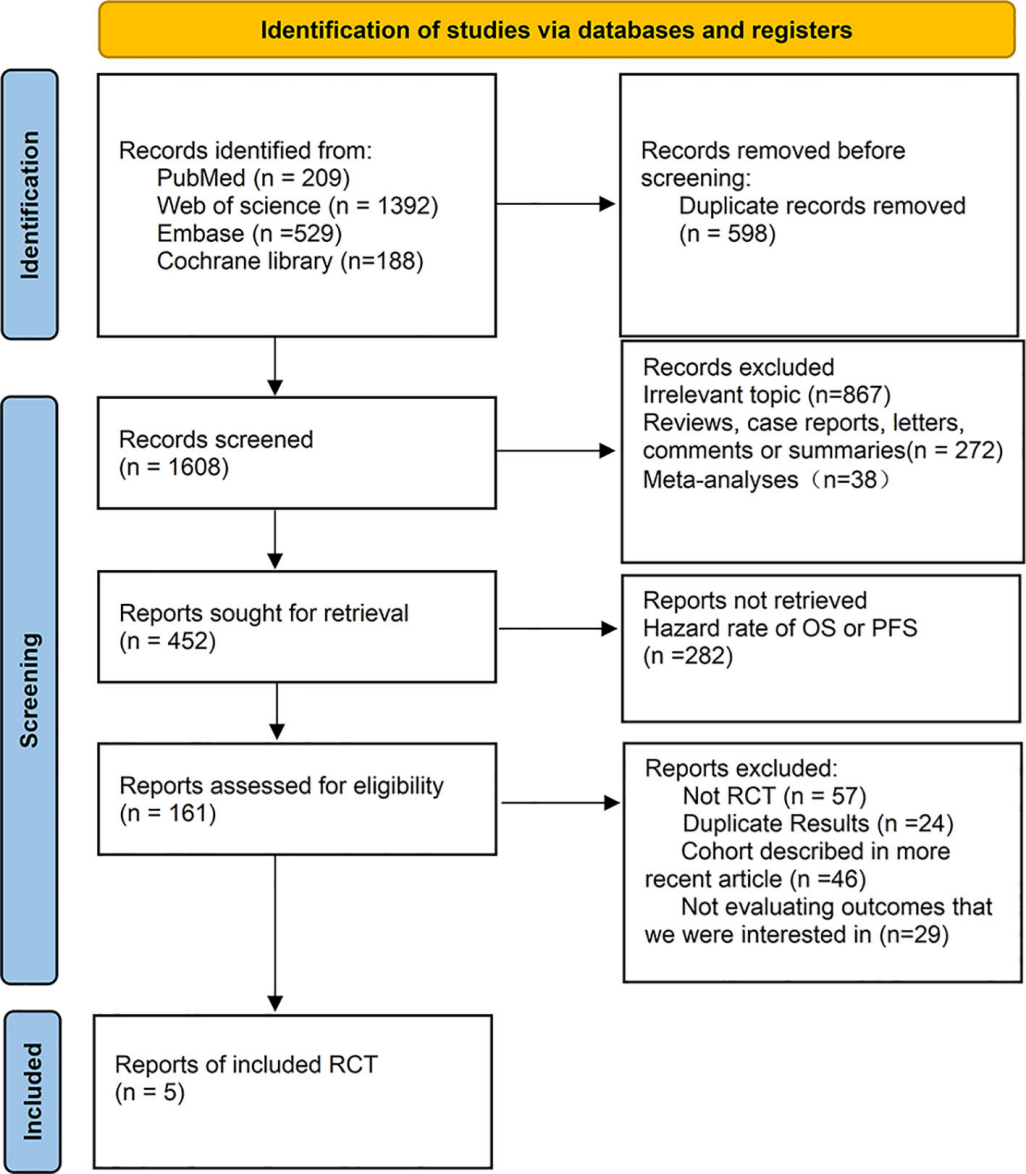
Flowchart of literature search and screening.

In the initial literature search for this systematic review, 1,401 records were retrieved from the databases. After screening abstracts to eliminate duplicates and irrelevant articles, 138 records met the criteria for full-text review. Ultimately, five studies fulfilled our eligibility criteria (**Figure 1**), involving a total of 2,078 patients who received the following second-line treatments: Sintilimab, Tislelizumab, Pembrolizumab, Nivolumab, Camrelizumab, and chemotherapy. Notably, the study involving Pembrolizumab included two types of esophageal cancer; we extracted data specifically for patients with advanced ESCC. Of these, Sintilimab and Camrelizumab were performed in China, and the other three were performed globally. Pembrolizumab and Tislelizumab used the Composite Positivity Score (CPS), which assesses PD-L1 expression by dividing the number of PD-L1-positive cells by the total number of tumor cells, and Camrelizumab uses the Tumor Proportion Score (TPS), which assesses PD-L1 expression by the fraction of positive tumor cells, and Tislelizumab uses the Tumor Area Positivity Score (TAP), which assesses PD-L1 expression by the area of PD-L1-stained tumor cells and immune cells as a percentage of the area of all tumors (38). The primary characteristics of these studies are detailed in **Tables 1**, **2**, and **Supplementary Table 4**, while the risk of bias assessment is shown in **Supplementary Figure 1**.

**Table 1:** Baseline characteristics of studies included in the network meta-analysis.

Study	Source	Registered ID	Sample Size	Histology	Ethnicity	Intervention Arm(s)	Control Arm(s)
Design	(Y)	(Randomization)	(Male/Female)				
ORIENT-2	Nat. Commun	NCT03116152	95/95	Advanced or metastatic esophageal squamous cell carcinoma	China	Sintilimab 200 mg once every 3 weeks	Paclitaxel was administered as 175 mg/m^2^ once every 3 weeks, or irinotecan was administered as180 mg/m^2^ once every 2 weeks
II	2022	(1:1)	172/18				
RATIONALE-302	J Clin Oncol	NCT03430843	256/256	Advanced or Metastatic Esophageal Squamous Cell Carcinoma	Asian	Tislelizumab 200 mg once every 3 weeks	Paclitaxel was administered as 135-175 mg/m^2^ IV once every 3 weeks, or in doses of 80-100 mg/m^2^ once weekly as per regional guidelines.
III	2022	(1:1)	432/80		Other		
KEYNOTE-181	J Clin Oncol	NCT02559687	314/314	Advanced Esophageal Cancer	Asian	Pembrolizumab 200 mg every 3 weeks	Chemotherapy with paclitaxel 80-100 mg/m^2^ on days 1, 8, and 15 of each 28-day cycle, docetaxel 75 mg/m^2^ on day 1 of each 21-day cycle, or irinotecan 180 mg/m^2^ on day 1 of each 14-day cycle
III	2020	(1:1)	544/84		Other		
ATTRACTION-3	Lancet Oncol	NCT02569242	210/209	Advanced oesophageal squamous cell carcinoma	Asian	Nivolumab was administered intravenously over 30 min at a dose of 240 mg every 2 weeks (each cycle was 6 weeks)	Paclitaxel and docetaxel were administered intravenously for at least 60 min; paclitaxel at 100 mg/m^2^ once per week for 6 weeks followed by 1 week off (each cycle was 7 weeks) and docetaxel at 75 mg/m^2^ every 3 weeks (each cycle was 3 weeks)
III	2019	(1:1)	364/55		Other		
ESCORT	Lancet Oncol	NCT03099382	228/220	Advanced or metastatic oesophageal squamous cell carcinoma	China	Camrelizumab was given intravenously over 30 min at a dose of 200 mg on day 1 of each 2-week cycle	Docetaxel (75 mg/m^2^, on day 1 of each 3-week cycle) or irinotecan (180 mg/m^2^, on day 1 of each 2-week cycle) were given intravenously over 60 min.

**Table 2:** Characteristics of included randomized controlled trials.

Study	PD-L1 Detection	PD-L1 ≥ 10% Patients (%)		PD-L1 < 10% Patients (%)		Reported Outcomes
		Intervention(s), n (%)	Control, n (%)	Intervention(s), n (%)	Control, n (%)	
ORIENT-2	CPS	40(42.1)	20(21.1)	34(35.8)	47(49.5)	OS, PFS, ORR, AEs, grade ≥ AEs
	TPS	14(14.7)	14(14.7)	60(63.2)	53(55.8)	
RATIONALE-302	TAP	89(34.8)	68(26.6)	116(45.3)	140(54.7)	OS, PFS, ORR, AEs, grade ≥ AEs
KEYNOTE-181	CPS	107(34.1)	115(36.6)	201(64)	196(62.4)	OS, PFS, ORR
ATTRACTION-3	CPS	64(30)	57(27)	146(70)	152(73)	OS, PFS, ORR, AEs, grade ≥ AEs
ESCORT	TPS	26(11)	35(16)	196(86)	181(82)	OS, PFS, ORR, AEs, grade ≥ AEs

### 3.2 Pairwise meta-analysis

#### 3.2.1 Comparisons of OS, PFS and ORR

**Figure 2:**
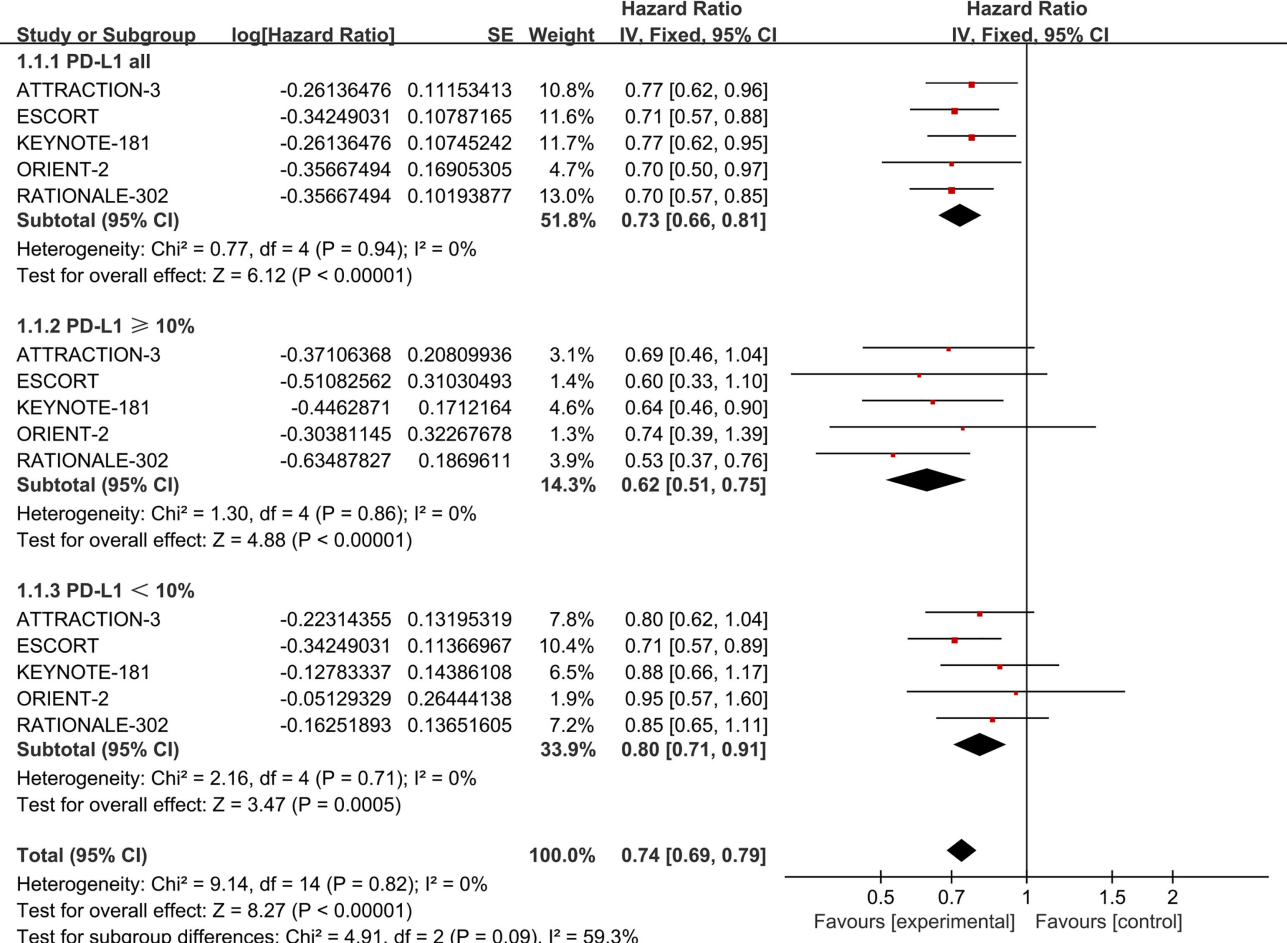
Forest plot comparing OS in ESCC patients with unselected PD-L1 expression (*P*=0.94, *I^2^* =0), PD-L1 ≥ 10% (*P*=0.86, *I^2^* =0), and PD-L1 < 10% (*P*=0.71, *I^2^* =0) receiving PD-1 inhibitors versus standard chemotherapy.

All five studies reported data on OS, PFS, and ORR. For OS, there was no statistically significant heterogeneity among the studies (*P* > 0.1, *I²* = 0). We therefore performed Meta-analysis using a fixed-effects model capable of assuming a true effect size for each comparison experiment. Patients with ESCC who did not have selected PD-L1 expression and were treated with PD-1 inhibitors demonstrated improved OS (HR = 0.73, 95% CI: 0.66-0.81) compared to those receiving standard chemotherapy. The results indicate that all PD-1 inhibitors significantly prolong OS compared to chemotherapy. Details are provided in **Figure 2**.

**Figure 3:**
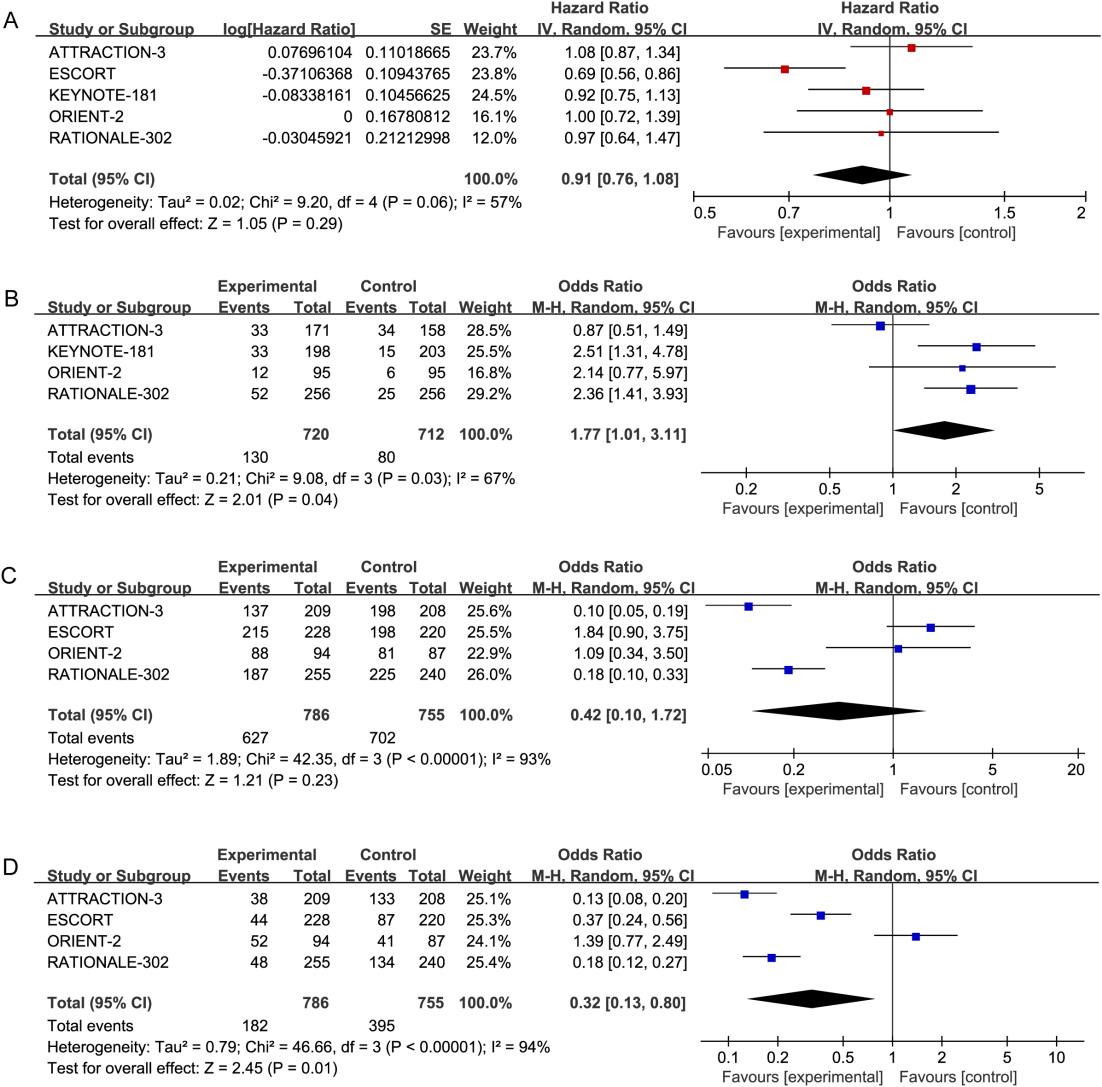
Forest plots: **(A)** PFS (*P*=0.06,*I^2^* =57%) in advanced ESCC patients receiving PD-1 inhibitors versus standard chemotherapy. **(B)** ORR (*P*=0.008, *I^2^* =71%) in advanced ESCC patients receiving PD-1 inhibitors versus standard chemotherapy. **(C)** Incidence of AEs (*P*<0.00001, *I^2^* =94%) in advanced ESCC patients receiving PD-L1inhibitors versus standard chemotherapy. **(D)** Incidence of Grade ≥AEs (*P*<0.00001, *I^2^* =92%) in advanced ESCC patients receiving PD-L1 inhibitors versus standard chemotherapy.

A random-effects model was applied to account for the possibility of varying true effect sizes across studies, as significant heterogeneity was observed among the five included studies (heterogeneity for PFS:*P* > 0.1, *I²* = 57%; heterogeneity for ORR: *P* > 0.1, *I²* = 71%), The results demonstrated that, in terms of PFS, the PD-1 inhibitor treatment group (HR = 0.91, 95% CI: 0.76-1.08) did not exhibit a significant improvement in PFS. Details are provided in **Figure 3A**.

Regarding ORR, Patients with ESCC receiving PD-1 inhibitors (OR = 2.07, 95% CI: 1.22-3.52) were more likely to experience an improvement in ORR compared to those receiving standard chemotherapy. These results indicate that PD-1 inhibitors significantly enhance ORR compared to chemotherapy. These results indicate that PD-1 inhibitors significantly enhance ORR compared to chemotherapy alone. Details are provided in **Figure 3B**.

#### 3.2.2 Safety and toxicity

The incidence of AEs and Grade ≥ 3 AEs was used to assess the safety and toxicity of PD-1 inhibitors. Five studies reported on AEs (*P* > 0.1, *I²* = 93%) and Grade ≥ 3 AEs (*P* > 0.1, *I²* = 94%), necessitating the use of a random-effects model for the meta-analysis. The results indicated no significant difference in the incidence of AEs among advanced ESCC patients receiving PD-1 inhibitors (OR = 0.42, 95% CI: 0.10-1.72). The incidence of Grade ≥ 3 AEs, however, was lower in patients treated with PD-1 inhibitors compared to those receiving standard chemotherapy (OR = 0.32, 95% CI: 0.13-0.80). Further details are presented in **Figures 3C, D**.

#### 3.2.3 Subgroup analysis

The study examined the OS outcomes of five PD-1 inhibitors across varying levels of PD-L1 expression. All five studies reported OS data for subgroups with PD-L1 ≥10% and PD-L1 <10%. Therefore, a subgroup analysis was performed on these two datasets.

No statistical heterogeneity was found among the studies (*P* > 0.1, *I²* = 0), supporting the use of a fixed-effect model in the meta-analysis. The results showed that ESCC patients with PD-L1 ≥ 10% (HR = 0.62, 95% CI: 0.51-0.75) and those with PD-L1 < 10% (HR = 0.80, 95% CI: 0.71-0.91) experienced significantly longer OS with PD-1 inhibitors compared to standard chemotherapy. Further details are provided in **Figure 2**.

### 3.3 Network meta-analyses

#### 3.3.1 Comparisons of OS, PFS and ORR

**Figure 4:**
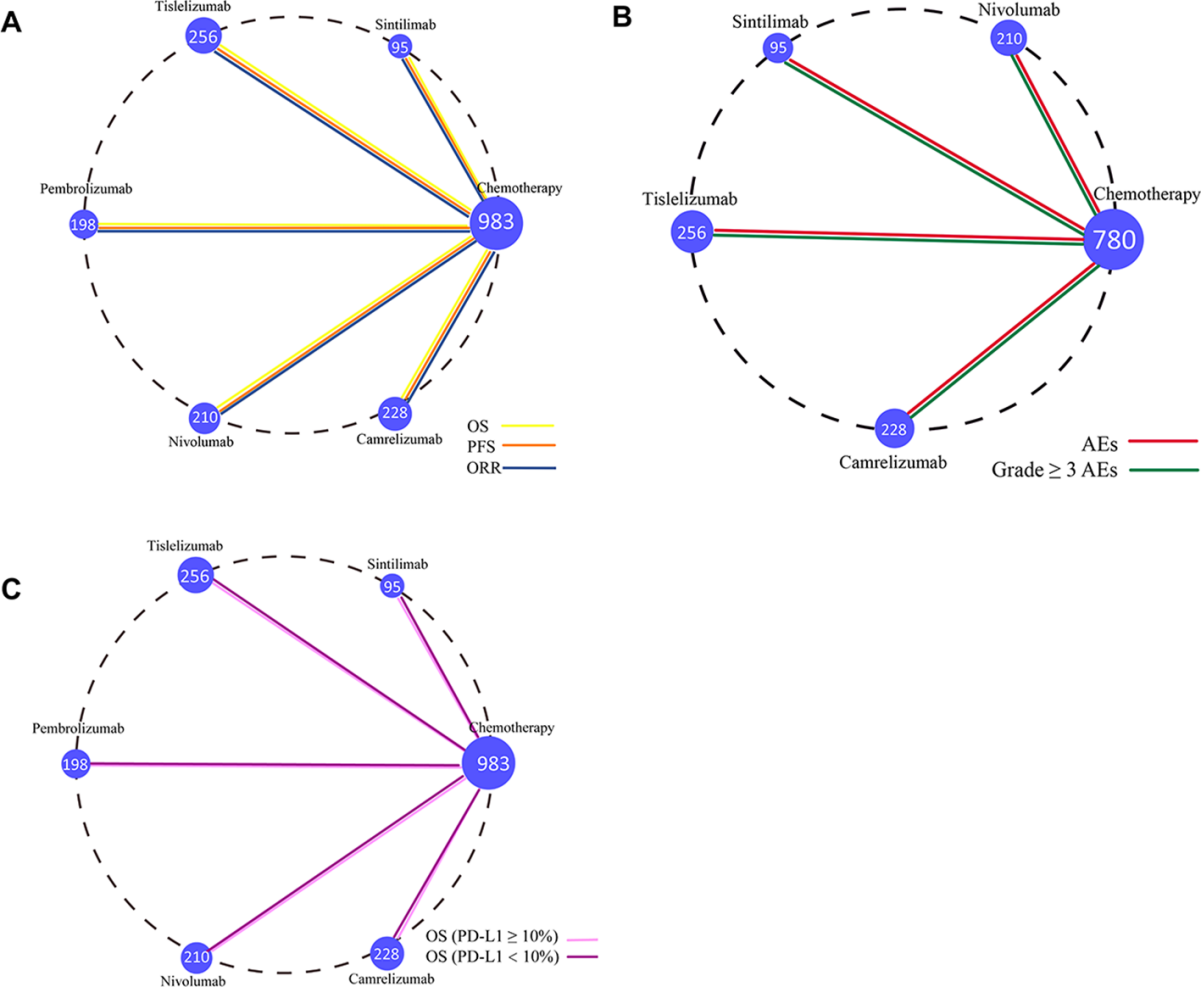
Network diagram comparing treatment outcomes in ESCC patients across different treatment groups. **(A)** Comparison of overall survival, progression-free survival, and objective response rate in ESCC patients. **(B)** Comparison of adverse event rates and Grade ≥ 3 AEs in ESCC patients. **(C)** Comparison of OS in ESCC patients with PD-L1 ≥ 10% and PD-L1 < 10%. Each node represents a treatment type, with the size of the node corresponding to the number of patients included. Two treatment regimens directly compared in a clinical trial are connected by a straight line.

The primary outcomes of this study—OS, PFS, and ORR—were used to evaluate treatment efficacy. The NMA included six treatment regimens for advanced ESCC patients, analyzing their OS, PFS, and ORR (**Figure 4A**).

**Figure 5:**
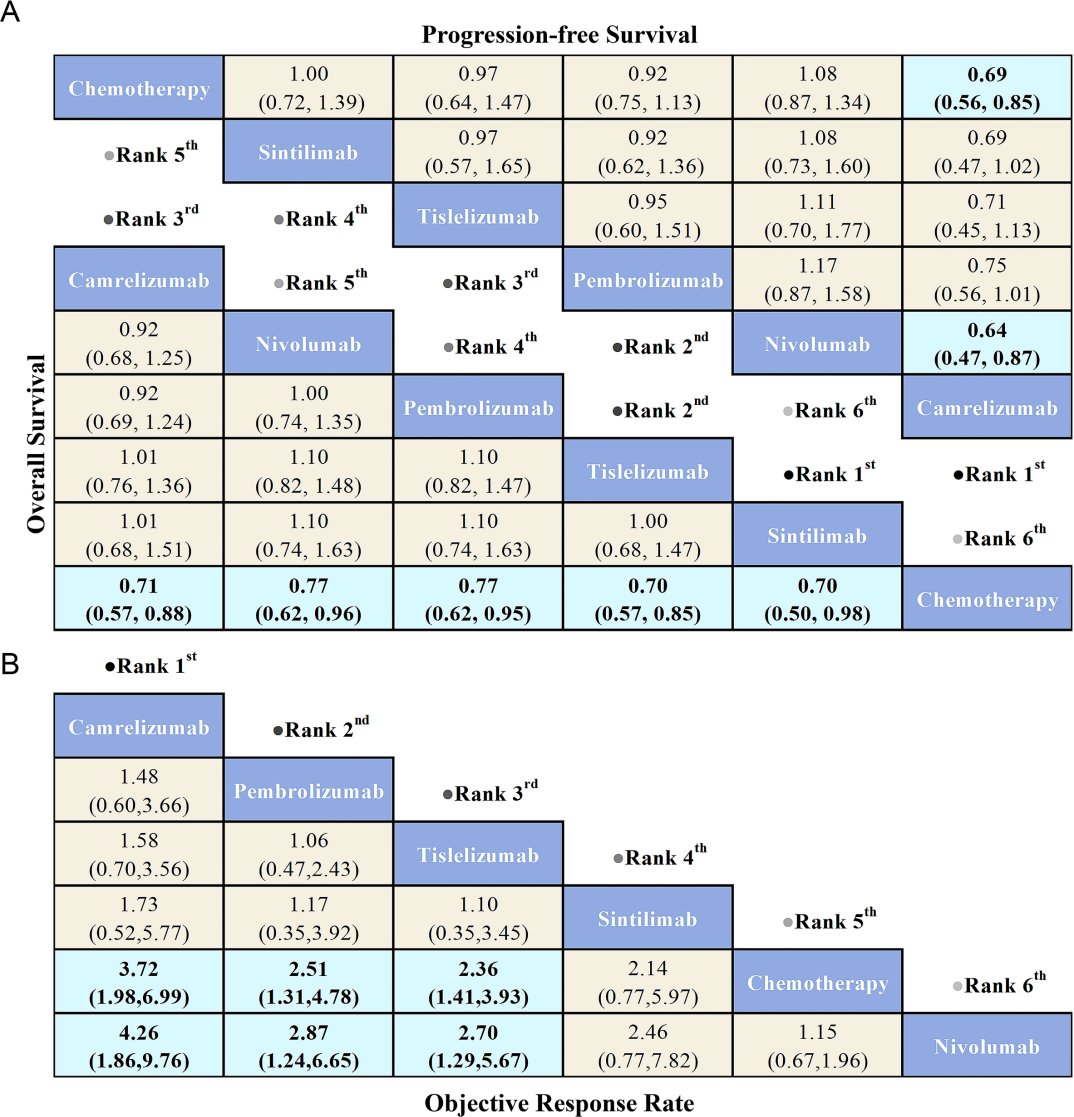
League table comparing the efficacy of various PD-1 inhibitors and chemotherapy in advanced ESCC patients based on Bayesian network meta-analysis. **(A)** The bottom-left triangular region shows the HRs and 95% CIs for overall survival, while the top-right triangular region shows HRs and 95% CIs for progression-free survival. An HR < 1.00 indicates better survival benefits. **(B)** The OR and 95% CIs for objective response rate, where an OR > 1.00 indicates better treatment benefits.

Regarding OS (**Figure 5A**), patients treated with PD-1 inhibitors were more likely to experience improved OS compared to those receiving chemotherapy. Among the PD-1 inhibitors, Sintilimab showed the greatest OS benefit (HR = 0.70, 95% CI: 0.50-0.98). Tislelizumab (HR = 0.70, 95% CI: 0.57-0.85) and Camrelizumab (HR = 0.71, 95% CI: 0.57-0.88) provided comparable OS benefits. Additionally, Pembrolizumab (HR = 0.77, 95% CI: 0.62-0.95) and Nivolumab (HR = 0.77, 95% CI: 0.62-0.96) also demonstrated similar OS advantages.

Regarding PFS (**Figure 5A**), PD-1 inhibitors did not show significant PFS benefits compared to chemotherapy, with the exception of Camrelizumab, which provided the greatest PFS advantage (HR = 0.69, 95% CI: 0.56-0.85). Notably, Nivolumab exhibited the poorest PFS among all treatment options, while Camrelizumab offered superior PFS compared to Nivolumab (HR = 0.64, 95% CI: 0.47-0.87).

Regarding ORR (**Figure 5B**), PD-1 inhibitors demonstrated superior ORR compared to chemotherapy, with the exception of Nivolumab, which showed the lowest ORR among all treatments. Camrelizumab provided the greatest ORR benefit relative to chemotherapy (OR = 3.72, 95% CI: 1.98-6.99), followed by Pembrolizumab (OR = 2.51, 95% CI: 1.31-4.78) and Tislelizumab (OR = 2.36, 95% CI: 1.41-3.93), both showing significant improvements in ORR. Additionally, Camrelizumab demonstrated a better ORR compared to Nivolumab (OR = 4.26, 95% CI: 1.86-9.76), with Pembrolizumab (OR = 2.87, 95% CI: 1.24-6.65) and Tislelizumab (OR = 2.70, 95% CI: 1.29-5.67) following closely.

#### 3.3.2 Safety and toxicity

The secondary outcomes of this study were AEs and Grade ≥ 3 AEs, which were used to assess safety and toxicity. To better interpret the ranking of adverse event occurrence rates, the results were reverse-ordered, meaning a higher ranking corresponds to a lower incidence of AEs. The NMA included five treatment regimens for advanced ESCC, focusing on AEs and Grade ≥ 3 AEs (**Figure 4B**).

**Figure 6:**
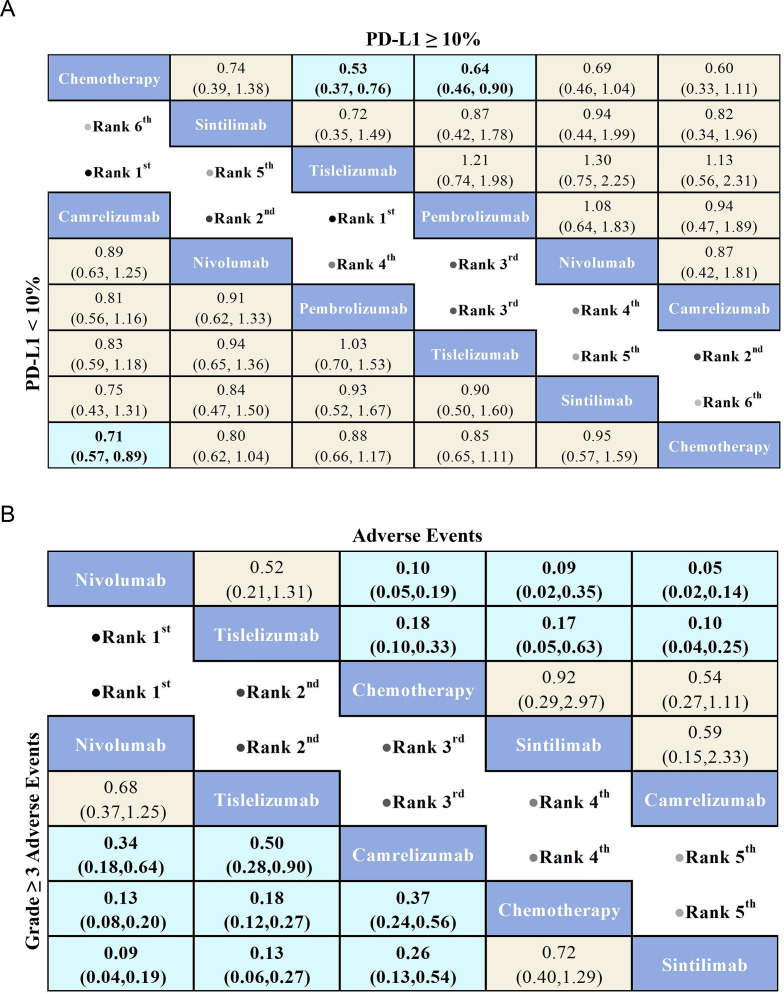
League table comparing the efficacy and safety of PD-1 inhibitors versus chemotherapy in advanced ESCC patients based on Bayesian network meta-analysis. **(A)** The top-right triangular region shows HRs and 95% CIs for overall survival in patients with PD-L1 ≥ 10%, while the bottom-left triangular region shows HRs and 95% CIs for those with PD-L1 < 10%. An HR < 1.00 indicates better survival benefits. **(B)** The top-right triangular region presents ORs and 95% CIs for adverse events, while the bottom-left triangular region shows ORs and 95% CIs for Grade ≥ 3 AEs. An OR < 1.00 indicates better safety.

Regarding AEs (**Figure 6B**), the Nivolumab group (OR = 0.10, 95% CI: 0.05-0.19) and Tislelizumab group (OR = 0.18, 95% CI: 0.10-0.33) demonstrated a significant safety advantage compared to chemotherapy. In contrast, Sintilimab (OR = 0.92, 95% CI: 0.29-2.97) and Camrelizumab (OR = 0.54, 95% CI: 0.27-1.11) did not show a significant safety benefit over chemotherapy. Notably, Nivolumab exhibited greater safety benefits compared to both Sintilimab (OR = 0.09, 95% CI: 0.02-0.35) and Camrelizumab (OR = 0.05, 95% CI: 0.02-0.14).

**Figure 7:**
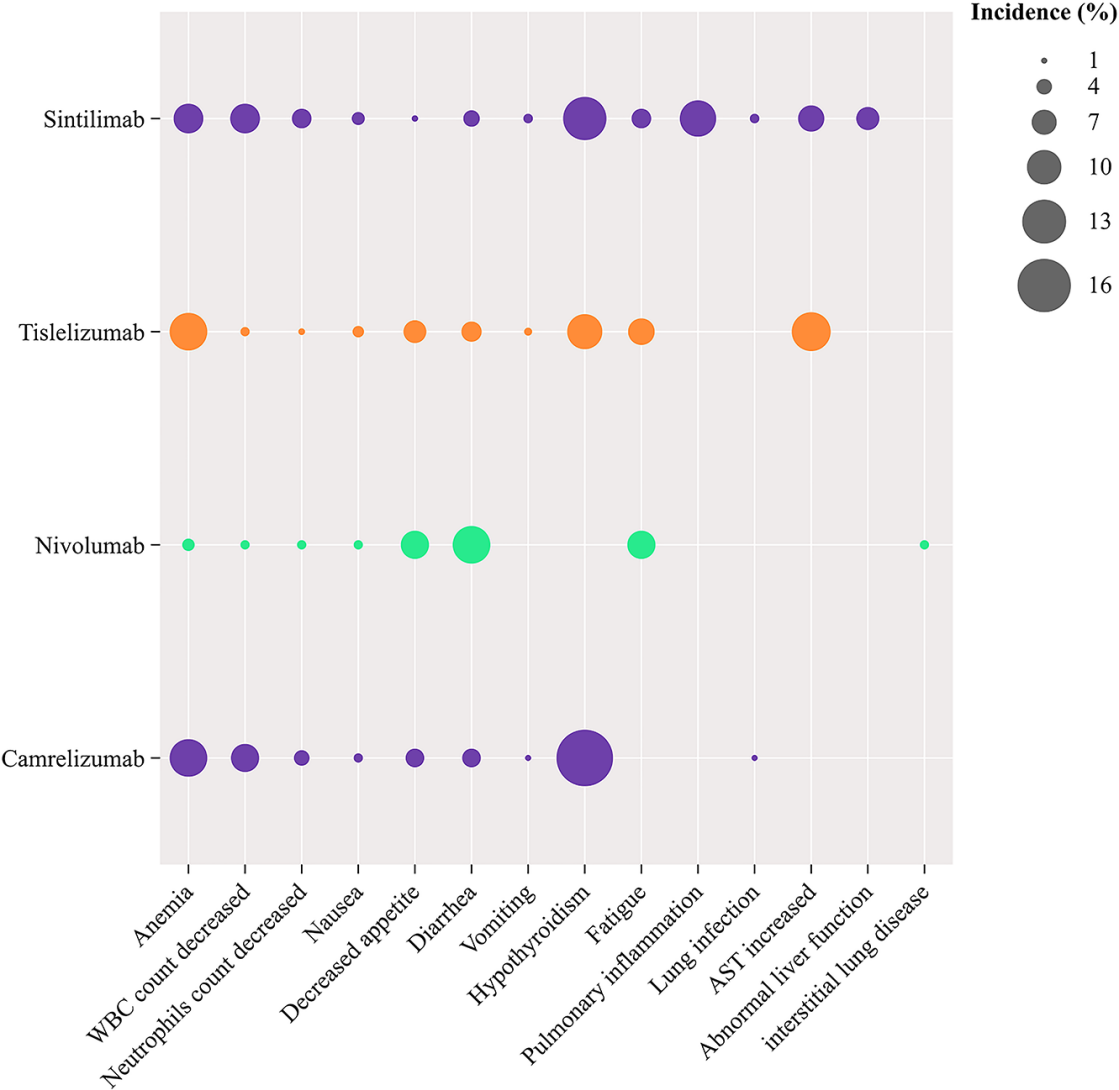
Bubble plot showing adverse events associated with PD-1 inhibitors.

Regarding Grade ≥ 3 AEs (**Figure 6B**), Nivolumab (OR = 0.13, 95% CI: 0.08-0.20) demonstrated a significant safety advantage over chemotherapy, with Tislelizumab (OR = 0.18, 95% CI: 0.12-0.27) and Camrelizumab (OR = 0.37, 95% CI: 0.24-0.56) also showing notable safety benefits compared to chemotherapy. In contrast, Sintilimab (OR = 0.72, 95% CI: 0.40-1.29) did not exhibit a significant safety advantage over chemotherapy. Additionally, Nivolumab (OR = 0.09, 95% CI: 0.04-0.19), Tislelizumab (OR = 0.13, 95% CI: 0.06-0.27), and Camrelizumab (OR = 0.26, 95% CI: 0.13-0.54) demonstrated greater safety benefits compared to Sintilimab. Commonly reported treatment-related adverse events for PD-1 inhibitors included anemia, nausea, decreased appetite, and reductions in white blood cell and neutrophil counts (**Figure 7**). Each PD-1 inhibitor exhibited varying rates of adverse events: The most common adverse events associated with Sintilimab were hypothyroidism, pneumonitis, anemia, and decreased white blood cell count. The most frequent severe adverse event was pneumonitis, with one fatal case each reported for upper gastrointestinal bleeding, pneumonia, and pulmonary infection.

The most common adverse events associated with Tislelizumab were elevated aspartate aminotransferase levels, anemia, and hypothyroidism. Fatal cases included one patient each for hemoptysis, pulmonary hypertension, upper respiratory tract infection, pneumonia, and thrombocytopenia, as well as three fatalities due to septic shock. The most common adverse events associated with Nivolumab were rash, diarrhea, and decreased appetite. The most frequent severe adverse events were fever and interstitial lung disease, with one fatal case each caused by interstitial lung disease and pneumonia, and the most common severe adverse events associated with Camrelizumab were anemia, liver dysfunction, and diarrhea. Fatal cases included one patient each due to colitis, liver dysfunction, pneumonia, and myocarditis. (**Supplementary Table S5**).

#### 3.3.3 Subgroup analysis

In the subgroup analysis, OS was used as the primary outcome measure. The NMA included six treatment regimens for advanced ESCC patients with varying PD-L1 expression levels (**Figure 4C**) to evaluate the efficacy of these treatments in patients with positive PD-L1 expression.

For OS in advanced ESCC patients with PD-L1 ≥ 10% (**Figure 6A**), five PD-1 inhibitors were included in the subgroup analysis. All treatment regimens provided an OS benefit compared to chemotherapy, with Tislelizumab (HR = 0.53, 95% CI: 0.37-0.76) being the most likely to offer the greatest OS benefit. Additionally, Pembrolizumab (HR = 0.64, 95% CI: 0.46-0.90) and Camrelizumab (HR=0.60,95%CI:0.33-1.11) also demonstrated a significant OS advantage over chemotherapy.

For OS in advanced ESCC patients with PD-L1 < 10% (**Figure 6A**), the results showed that PD-1 inhibitor treatments provided a clear OS benefit over standard chemotherapy. Among the treatments, Camrelizumab (HR = 0.71, 95% CI: 0.57-0.89) was the most likely to offer the greatest OS benefit. Additionally, Nivolumab (HR=0.80,95%CI:0.62-1.04) and Tislelizumab (HR=0.85,95%CI:0.65-1.11) also demonstrated a significant OS advantage over chemotherapy.

#### 3.3.4 Ranking

**Figure 8:**
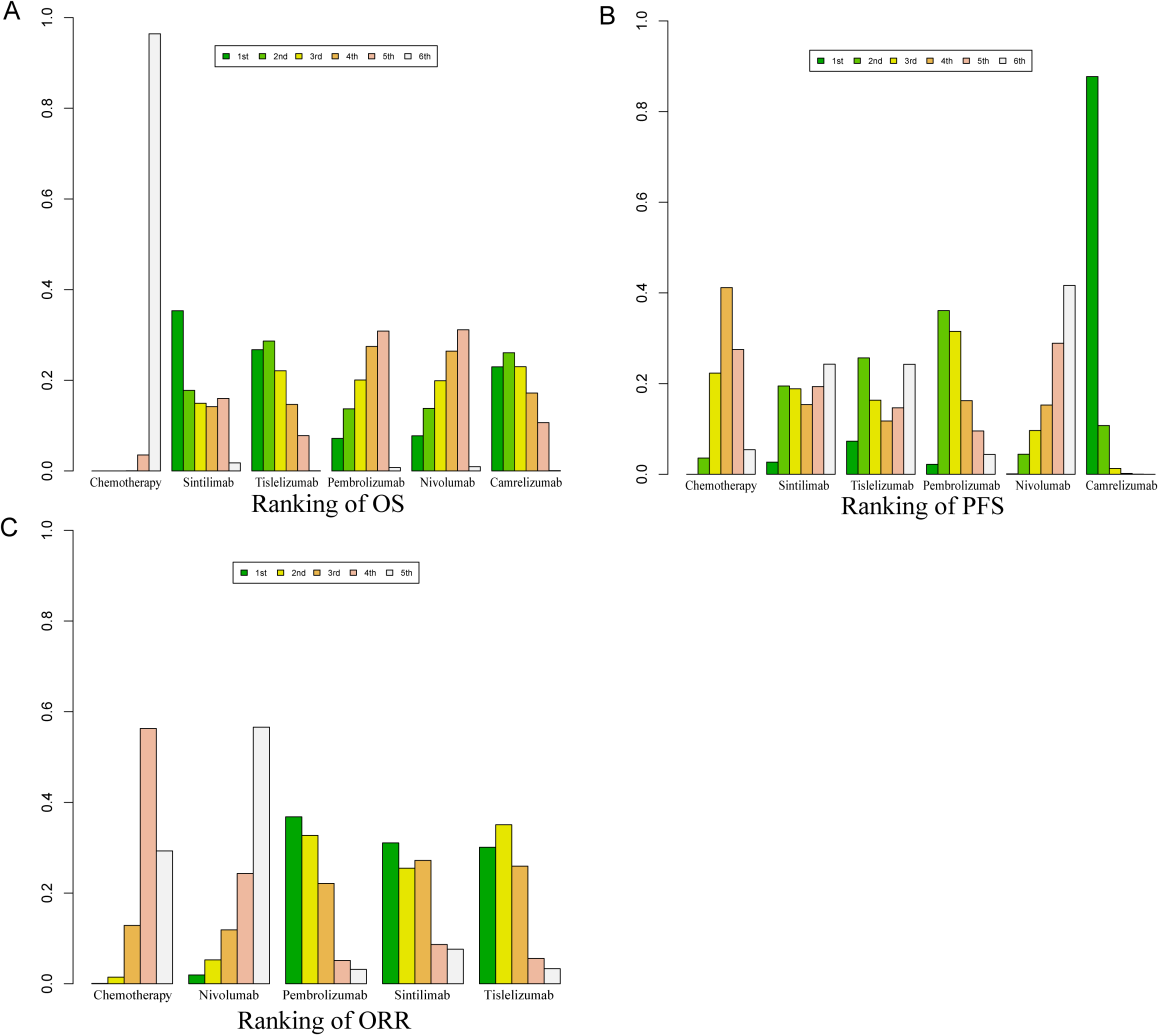
Bayesian ranking profile for the efficacy of various PD-1 inhibitor treatment regimens in advanced ESCC patients. **(A)** Ranking of OS. **(B)** Ranking of PFS. **(C)** Ranking of ORR.

**Figure 9:**
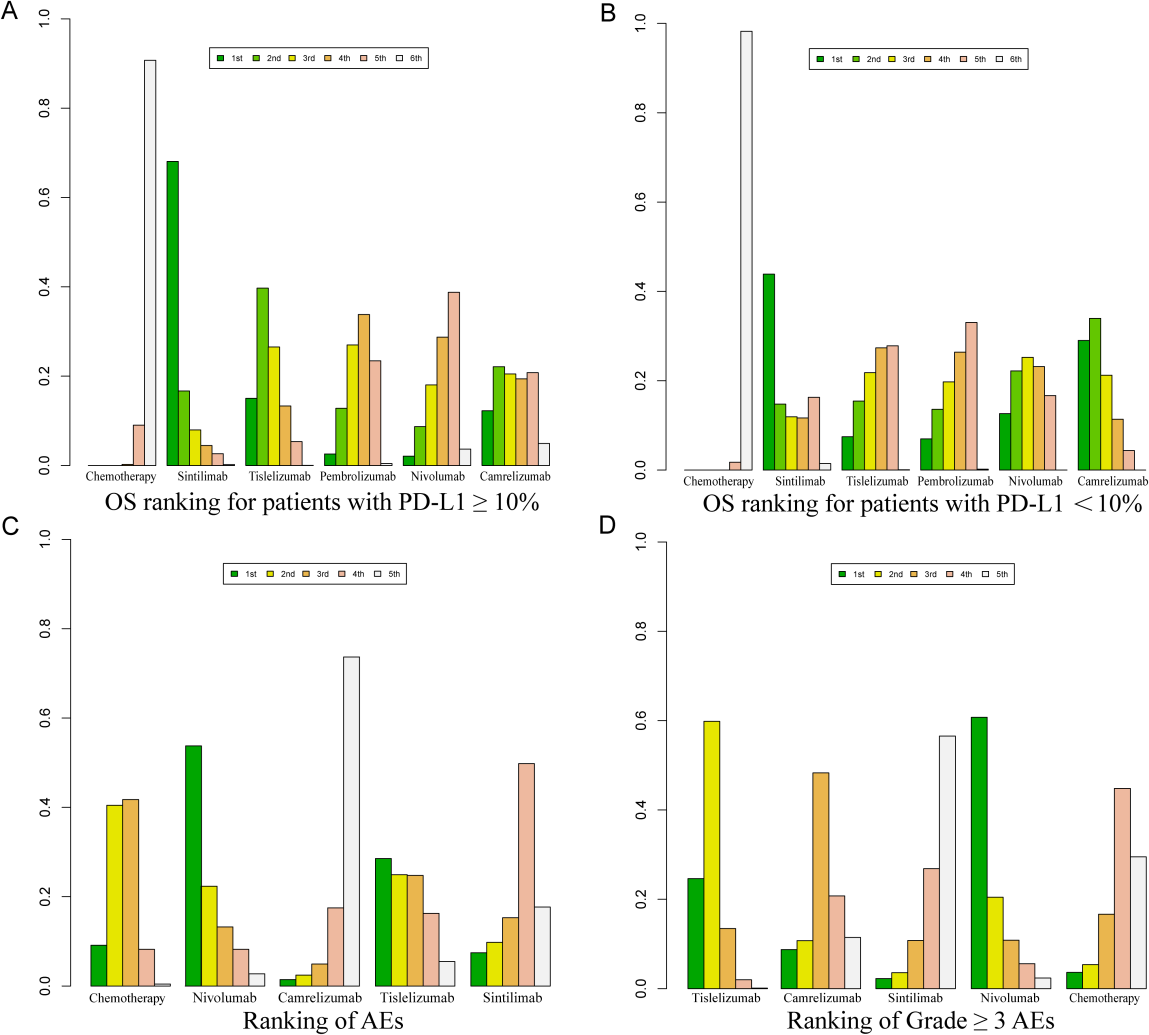
Bayesian ranking profile for the efficacy and safety of various PD-1 inhibitor treatment regimens in advanced ESCC patients. **(A)** OS ranking for patients with PD-L1 ≥ 10%. **(B)** OS ranking for patients with PD-L1 < 10%. **(C)** Ranking of AEs. **(D)** Ranking of Grade ≥ 3 AEs.

Bayesian ranking curve analysis (**Figures 8**, **9**, **Supplementary Tables S6**–**Supplementary Table S10**) was performed to assess the ranking probabilities of the six treatment regimens included in the study. For patients without selected PD-L1 expression, Sintilimab ranked first for OS, with a probability of 35.19%. Among patients with PD-L1 ≥ 10%, Tislelizumab ranked first for OS (probability of 43.56%), followed by Camrelizumab in second place (probability of 28.28%). For patients with PD-L1 < 10%, Camrelizumab ranked first for OS, with a probability of 55.63%.

For PFS, Camrelizumab ranked first with a probability of 87.74%, and for ORR, it also ranked first with a probability of 40.40%. Regarding AEs of any grade, Nivolumab ranked first with a probability of 91.70%, and for Grade ≥ 3 AEs, Nivolumab again ranked first with a probability of 89.3%.

Tislelizumab achieved a balance between efficacy and safety. It ranked second for OS in patients without selected PD-L1 expression (probability of 28.40%), first for OS in patients with PD-L1 ≥ 10% (probability of 43.56%), and third for OS in patients with PD-L1 < 10% (probability of 26.27%). In PFS, Tislelizumab ranked third (probability of 16.35%), in ORR it ranked third (probability of 41%), second for AEs (probability of 91.30%), and second for Grade ≥ 3 AEs (probability of 88.3%).

#### 3.3.5 Assessment of heterogeneity, inconsistency, and transitivity

The pairwise meta-analysis results using the frequentist approach aligned with the corresponding summaries from the Bayesian framework (**Supplementary Table 3**). Heterogeneity was assessed using the Q test and *I²* statistic, with results indicating high heterogeneity (*I²* > 50%) (**Figures 2**–**4**). Sequential exclusion of individual studies did not significantly reduce the heterogeneity. All RCTs involved comparisons between a single PD-1 inhibitor and chemotherapy, eliminating the possibility of inconsistency. Therefore, an inconsistency check was not performed (39). A funnel plot analysis was performed to evaluate publication bias, with OS as the outcome measure. The symmetrical distribution of scatter points, with no outliers detected, indicates a low probability of publication bias in this study (**Supplementary Figure 1**).

## 4 Discussion

### 4.1 Principal findings

To the best of our knowledge, this is the most comprehensive systematic review and NMA to date, comparing the efficacy and safety of second-line PD-1 inhibitors. It also includes an evaluation of OS outcomes in both PD-L1 ≥ 10% and PD-L1 < 10% populations. This study offers evidence-based insights to inform clinical practice and highlights the following key findings:

1. All immunotherapy regimens demonstrated superior OS and ORR compared to standard chemotherapy.
2. PD-1 inhibitors did not show a significant advantage in PFS over chemotherapy, with the exception of Camrelizumab, which provided a distinct PFS benefit.
3. For advanced ESCC patients with PD-L1 ≥ 10%, Tislelizumab was associated with the most pronounced OS benefit, whereas Camrelizumab was most likely to yield the greatest OS improvement for patients with PD-L1 <10%. (4) The safety profiles of different PD-1 inhibitors varied significantly compared to standard chemotherapy. Nivolumab (OR = 0.10, 95% CI: 0.05-0.19) and Tislelizumab (OR = 0.18, 95% CI: 0.10-0.33) demonstrated better safety in terms of AEs, while Nivolumab (OR = 0.10, 95% CI: 0.05-0.19) showed superior safety in terms of Grade ≥ 3 AEs compared to chemotherapy.

The PD-L1/PD-1 pathway acts as a synergistic inhibitory signaling mechanism, in which PD-L1 binds to its receptor PD-1 on activated T cells, preserving peripheral T cell tolerance and maintaining immune homeostasis to prevent overactivation and autoimmune disorders (40). In malignancies, the interaction between PD-L1 and PD-1 suppresses T cell activation, enabling tumors to evade anti-tumor immunity (41). Additionally, PD-L1 is frequently overexpressed in cancer cells, with its production triggered by various cytokines within the tumor microenvironment (42). Consequently, PD-1 inhibitors demonstrate anti-tumor activity by blocking the PD-L1/PD-1 pathway and reactivating T cell immune function (16, 43). In contrast, chemotherapy agents elicit specific immune responses against tumors through the induction of immunogenic cell death (44, 45). Taxane-based therapies are widely utilized as second-line treatments following first-line failure, with OS typically ranging from 8 to 10 months (46). With the remarkable success of immunotherapy clinical trials, numerous studies have investigated the use of immunotherapy in advanced ESCC. Notably, several phase III trials, including ESCORT-1 (47, 48), have successfully established PD-1 inhibitors as key treatments for advanced ESCC.

This study conducted a statistical analysis of AEs and Grade ≥ 3 AEs, with no new safety events identified. The occurrence of AEs with different PD-1 inhibitors was consistent with the findings of Wang’s meta-analysis (49). However, unlike Wang’s study, our analysis provides additional data on the specific probabilities of adverse events associated with each PD-1 inhibitor.

Notably, compared to chemotherapy, PD-1 inhibitors significantly improved OS and ORR in second-line treatment for ESCC, although the difference in PFS was less pronounced. This may be attributed to the longer duration of immunotherapy.

Challenges in early diagnosis and accurate prognosis for ESCC patients have hindered effective treatment of this disease. Identifying biomarkers for clinical diagnosis and prognosis of ESCC remains an urgent priority in this field (50, 51). PD-L1 expression levels are used as a biomarker to predict the clinical efficacy of immunotherapy, a meta-analysis indicated an association between PD-L1 expression and OS in ESCC (52). The meta-analyses by Wang (53) and Zhu (28) focused solely on comparing the OS benefits of individual PD-1 inhibitors between patients with high and low PD-L1 expression and chemotherapy. However, they did not conduct an in-depth analysis of PD-1 inhibitor treatment in PD-L1-positive advanced ESCC patients. This study stratified patients based on PD-L1 expression levels and found that, except for the Sintilimab group, advanced ESCC patients with PD-L1 ≥10% showed greater OS benefits compared to those without PD-L1 expression selection. Additionally, the OS benefits of different PD-1 inhibitors were compared between the PD-L1 ≥10% and PD-L1 <10% subgroups, identifying the optimal immunotherapy regimens for each PD-L1 expression subgroup.

### 4.2 Implications

A comprehensive meta-analysis of the most robust randomized controlled trial data to evaluate the efficacy and safety of second-line PD-1 inhibitors, stratified by PD-L1 expression levels, offering valuable insights for clinical decision-making. Considering both clinical efficacy and safety, Tislelizumab was identified as a promising second-line immunotherapy option for advanced ESCC patients without selected PD-L1 expression. Additionally, the findings show that patients with PD-L1 ≥ 10% experience improved survival outcomes with Tislelizumab. This could contribute to refining clinical guidelines regarding the selection of PD-1 inhibitors for second-line immunotherapy in advanced ESCC patients. Specifically, for advanced ESCC patients with PD-L1 expression levels ≥10%, we recommend considering Tislelizumab as the preferred treatment option.

### 4.3 Limitations

While this study draws several important conclusions, it also has the following limitations. First, PD-L1 expression is anticipated to serve as a crucial biomarker in the development of immune checkpoint inhibitors, aiding in the precise selection of treatment strategies and providing prognostic information (54). However, there are significant differences in how PD-L1 is evaluated across various PD-1 inhibitors. For instance, Pembrolizumab and Nivolumab use the Combined Positive Score (CPS) to assess PD-L1 expression in advanced ESCC patients, while Camrelizumab uses the Tumor Proportion Score (TPS). In March 2024, Tislelizumab was approved by the FDA for the treatment of advanced ESCC and employs the Tumor Area Positive Score (TAP) to evaluate PD-L1 expression in these patients. The differences in PD-L1 expression can be attributed to variations in detection methods, resulting in discrepancies in PD-L1 staining outcomes. Additionally, PD-L1 expression may fluctuate over time and across tumor sites, further influencing evaluators’ assessments and contributing to intergroup heterogeneity in PD-L1 expression. Additionally, the clinical utility of biomarkers such as CPS and TPS remains uncertain, which may introduce bias when comparing PD-L1 expression outcomes across different PD-1 inhibitor trials in the second-line treatment of locally advanced ESCC. Among the five PD-1 inhibitors, only Camrelizumab and Nivolumab explored PD-L1 expression levels beyond the PD-L1 ≥10% and PD-L1 <10% subgroups, including PD-L1 ≥1%, PD-L1 <1%, PD-L1 ≥5%, and PD-L1 <5%. Consequently, our analysis of OS outcomes in patients with different PD-L1 expression levels is limited to the PD-L1 ≥10% and PD-L1 <10% subgroups. This limitation may also affect the evaluation of different PD-1 inhibitors in treating advanced ESCC patients with varying PD-L1 expression levels.

Second, ESCC was found to exhibits significant regional heterogeneity, with notable biological and clinical differences between Eastern and Western patients, which presents challenges for clinical research (55). For instance, the KEYNOTE-181 trial conducted a subgroup analysis of advanced ESCC patients, dividing them into an Asian subgroup and a Western subgroup. The analysis revealed that the Asian subgroup experienced greater benefits, suggesting that patients from different regions may derive varying levels of benefit. Consequently, the applicability of these findings to other ethnic groups remains uncertain and warrants further discussion. This regional heterogeneity may also influence our analysis of the benefits of PD-1 inhibitors in the second-line treatment of advanced ESCC.

Third, immune checkpoint inhibitor monotherapy was observed to be better tolerated, with a lower incidence of AEs and significantly fewer Grade ≥ 3 AEs compared to chemotherapy (27, 53). However, due to their distinct mechanism of action,immune checkpoint inhibitors are associated with specific adverse events, predominantly affecting the endocrine system, gastrointestinal tract, lungs, and skin (56, 57). It is important to note that serious immune-related adverse events (IRAEs) may develop in a small subset of patients, and these reactions can lead to severe outcomes, such as high mortality rates from cardiovascular complications induced by immune checkpoint inhibitor therapy. Unfortunately, most studies involving immune checkpoint inhibitors do not clearly differentiate between IRAEs and common AEs. This limits our ability to comprehensively assess the safety profile of immune checkpoint inhibitors in the treatment of advanced ESCC.

Fourth, as our NMA included only five RCTs with a relatively small number of trials and participants, this may impact the validity of the Bayesian analysis results. Additionally, since the ROB 2.0 tool lacks specific evaluation criteria related to data bias, we remain concerned about the reliability of the bias assessment outcomes (58). In the future, more refined literature evaluation tools will be needed to study the efficacy and safety of PD-1 inhibitors in treating advanced ESCC patients, providing better guidance for clinical decision-making.

Fifth, as second-line treatment with PD-1 inhibitors exclusively involves single-agent regimens, our discussion focuses solely on the efficacy and safety of single-agent PD-1 inhibitors for ESCC as second-line therapy, without addressing combination immunotherapy. While PD-1 inhibitors demonstrate better efficacy and safety compared to chemotherapy, they still have limitations, including immune-related adverse events and poor tumor tissue penetration. Future efforts could focus on developing PD-L1 small-molecule drugs to reduce attacks on normal cells and minimize immune-related adverse events. Alternatively, combining PD-1 inhibitors with targeted therapies could enhance drug delivery directly to tumor tissues, achieving more effective treatment outcomes (53).

Despite these limitations, this study offers a thorough summary of randomized controlled trials evaluating second-line immunotherapy for patients with advanced ESCC. This also provides a valuable reference for the treatment of advanced ESCC patients with varying PD-L1 expression levels using PD-1 inhibitors.

## Data Availability

The original contributions presented in the study are included in the article/**Supplementary Material**. Further inquiries can be directed to the corresponding authors.
