# Supplementary material for: Efficacy and safety of PD-1 inhibitors as second-line treatment for advanced squamous esophageal cancer: a systematic review and network meta-analysis with a focus on PD-L1 expression levels
Source: Front Immunol. 2025 Jan 23;15:1510145. doi: 10.3389/fimmu.2024.1510145 (PMC11798917; doi:10.3389/fimmu.2024.1510145)
Supplement: Supplementary file 1 [file Table1.docx]

| **Table of Contents** | | |
| --- | --- | --- |
| **Title** | **Contents** | **Page** |
| Table S1 | PRISMA NMA Checklist of Items to Include When Reporting a Systematic Review Involving a Network Meta-analysis | 2-4 |
| Table S2 | Literature Search Strategy | 5-8 |
| Table S3 | Results of Head-to-head Meta-analysis According to Pairwise Meta-analysis and Comparisons Between bayesian Network Meta-analysis | 9 |
| Table S4 | baseline Clinical and disease Characteristics of Trials Included in the Network Meta-analysis | 10-12 |
| Table S5 | Incidence of Treatment-related Adverse Events in Each Immunotherapy Regimen | 13 |
| Table S6 | Ranking Profiles in the Bayesian Network Meta-analysis (Overall Survival) | 14 |
| Table S7 | Ranking Profiles in the Bayesian Network Meta-analysis (Progression-free Survival) | 15 |
| Table S8 | Ranking Profiles in the Bayesian Network Meta-analysis (Objective Response Rate) | 16 |
| Table S9 | Ranking Profiles in the Bayesian Network Meta-analysis (Adverse Events) | 17 |
| Table S10 | Ranking Profiles in the Bayesian Network Meta-analysis (grade ≥ 3 AEs) | 18 |
| Figure S1 | Summary of results from assessment of studies by using Cochrane Risk of Bias Tool 2.0 | 19 |
| Figure S2 | Funnel plot to detect the publication bias of included studies | 20 |

**Table S1. PRISMA NMA Checklist of Items to Include When Reporting A Systematic Review Involving a Network Meta-analysis.**

| **Section/Topic** | **Item #** | **Checklist Item** | **Reported on Page #** |
| --- | --- | --- | --- |
| **TITLE** |  |  |  |
| Title | 1 | Identify the report as a systematic review *incorporating*  *anetwork meta-analysis (or related form of meta-analysis).* | **1** |
|  |  |  |  |
| **ABSTRACT** |  |  |  |
| Structured summary | 2 | Provide a structured summary including, as applicable:  **Background:** main objectives  **Methods:** data sources; study eligibility criteria, participants, and interventions; study appraisal; and *synthesis methods, such as network meta-analysis.*  **Results:** number of studies and participants identified; summary estimates with corresponding confidence/credible intervals; *treatment rankings may also be discussed. Authors may choose to summarize pairwise comparisons against a chosen treatment included in their analyses for brevity.*  **Discussion/Conclusions:** limitations; conclusions and implications of findings.  **Other:** systematic review registration number with registry name. | 1-2 |
|  |  |  |  |
| **INTRODUCTION** |  |  |  |
| Rationale | 3 | Describe the rationale for the review in the context of what is already known*, including mention of why a network meta-analysis has been conducted.* | **2-3** |
| Objectives | 4 | Provide an explicit statement of questions being addressed, with reference to participants, interventions, comparisons, outcomes, and study design (PICOS). | 3 |
|  |  |  |  |
| **METHODS** |  |  |  |
| Protocol and registration | 5 | Indicate whether a review protocol exists and if and where it can be accessed (e.g., Web address); and, if available, provide registration information, including registration number. | **3** |
| Eligibility criteria | 6 | Specify study characteristics (e.g., PICOS, length of follow-up) and report characteristics (e.g., years considered, language, publication status) used as criteria for eligibility, giving rationale. *Clearly describe eligible treatments included in the treatment network, and note whether any have been clustered or merged into the same node (with justification).* | 3-4, Table 1 |
| Information sources | 7 | Describe all information sources (e.g., databases with dates of coverage, contact with study authors to identify additional studies) in the search and date last searched. | **3** |
| Search | 8 | Present full electronic search strategy for at least one database, including any limits used, such that it could be repeated. | 3，Supplementary  TableS2 |
| Study selection | 9 | State the process for selecting studies (i.e., screening, eligibility, included in systematic review, and, if applicable, included in the meta-analysis). | **4** |
| Data collection process | 10 | Describe method of data extraction from reports (e.g., piloted forms, independently, in duplicate) and any processes for obtaining and confirming data from investigators. | 4 |
| Data items | 11 | List and define all variables for which data were sought (e.g., PICOS, funding sources) and any assumptions and simplifications made. | **5** |
| **Geometry of the network** | **S1** | Describe methods used to explore the geometry of the treatment network under study and potential biases related to it. This should include how the evidence base has been graphically summarized for presentation, and what characteristics were compiled and used to describe the evidence base to readers. | 5 |
| Risk of bias within individual studies | 12 | Describe methods used for assessing risk of bias of individual studies (including specification of whether this was done at the study or outcome level), and how this information is to be used in any data synthesis. | **5** |
| Summary measures | 13 | State the principal summary measures (e.g., risk ratio, difference in means). *Also describe the use of additional summary measures assessed, such as treatment rankings and surface under the cumulative ranking curve (SUCRA) values, as well as modified approaches used to present summary findings from meta-analyses.* | 5-6 |
| Planned methods of analysis | 14 | Describe the methods of handling data and combining results of studies for each network meta-analysis. This should include, but not be limited to:   - *Handling of multi-arm trials;* - *Selection of variance structure;* - *Selection of prior distributions in Bayesian analyses; and* - *Assessment of model fit.* | **5-6** |
| **Assessment of Inconsistency** | **S2** | Describe the statistical methods used to evaluate the agreement of direct and indirect evidence in the treatment network(s) studied. Describe efforts taken to address its presence when found. | 5-6 |
| Risk of bias across studies | 15 | Specify any assessment of risk of bias that may affect the cumulative evidence (e.g., publication bias, selective reporting within studies). | **5** |
| Additional analyses | 16 | Describe methods of additional analyses if done, indicating which were pre-specified. This may include, but not be limited to, the following:   - Sensitivity or subgroup analyses; - Meta-regression analyses; - *Alternative formulations of the treatment network; and* - *Use of alternative prior distributions for Bayesian analyses (if applicable).* | 5-6 |
|  |  |  |  |
| **RESULTS** |  |  |  |
| Study selection | 17 | Give numbers of studies screened, assessed for eligibility, and included in the review, with reasons for exclusions at each stage, ideally with a flow diagram. | **6** |
| **Presentation of network structure** | **S3** | Provide a network graph of the included studies to enable visualization of the geometry of the treatment network. | 15 |
| **Summary of network geometry** | **S4** | Provide a brief overview of characteristics of the treatment network. This may include commentary on the abundance of trials and randomized patients for the different interventions and pairwise comparisons in the network, gaps of evidence in the treatment network, and potential biases reflected by the network structure. | **5，10-22** |
| Study characteristics | 18 | For each study, present characteristics for which data were extracted (e.g., study size, PICOS, follow-up period) and provide the citations. | Table 1, Table 2, Supplementary Table 4 |
| Risk of bias within studies | 19 | Present data on risk of bias of each study and, if available, any outcome level assessment. | **Supplementary Figure S1** |
| Results of individual studies | 20 | For all outcomes considered (benefits or harms), present, for each study: 1) simple summary data for each intervention group, and 2) effect estimates and confidence intervals. *Modified approaches may be needed to deal with information from larger networks.* | Table 1, Table 2, Supplementary Table 4 |
| Synthesis of results | 21 | Present results of each meta-analysis done, including confidence/credible intervals. *In larger networks, authors may focus on comparisons versus a particular comparator (e.g. placebo or standard care), with full findings presented in an appendix. League tables and forest plots may be considered to summarize pairwise comparisons.* If additional summary measures were explored (such as treatment rankings), these should also be presented. | **10-22** |
| **Exploration for inconsistency** | **S5** | Describe results from investigations of inconsistency. This may include such information as measures of model fit to compare consistency and inconsistency models, *P* values from statistical tests, or summary of inconsistency estimates from different parts of the treatment network. | 22 |
| Risk of bias across studies | 22 | Present results of any assessment of risk of bias across studies for the evidence base being studied. | **22, Supplementary Figure S2** |
| Results of additional analyses | 23 | Give results of additional analyses, if done (e.g., sensitivity or subgroup analyses, meta-regression analyses*, alternative network geometries studied, alternative choice of prior distributions for Bayesian analyses,* and so forth). | 16-17 |
|  |  |  |  |
| **DISCUSSION** |  |  |  |
| Summary of evidence | 24 | Summarize the main findings, including the strength of evidence for each main outcome; consider their relevance to key groups (e.g., healthcare providers, users, and policy-makers). | **23-24** |
| Limitations | 25 | Discuss limitations at study and outcome level (e.g., risk of bias), and at review level (e.g., incomplete retrieval of identified research, reporting bias). *Comment on the validity of the assumptions, such as transitivity and consistency. Comment on any concerns regarding network geometry (e.g., avoidance of certain comparisons).* | 24-25 |
| Conclusions | 26 | Provide a general interpretation of the results in the context of other evidence, and implications for future research. | **25** |
|  |  |  |  |
| **FUNDING** |  |  | Na |
| Funding | 27 | Describe sources of funding for the systematic review and other support (e.g., supply of data); role of funders for the systematic review. This should also include information regarding whether funding has been received from manufacturers of treatments in the network and/or whether some of the authors are content experts with professional conflicts of interest that could affect use of treatments in the network. |  |

PICOS = population, intervention, comparators, outcomes, study design.

* Text in italics indicate S wording specific to reporting of network meta-analyses that has been added to guidance from the PRISMA statement.

† Authors may wish to plan for use of appendices to present all relevant information in full detail for items in this section.

Table S2. Literature Search Strategy

| **Table S1. Literature Search Strategy** | |
| --- | --- |
| **Pubmed** | (((((((((((((((((((Esophageal Neoplasms[MeSH Terms])) OR (esophageal neoplasm[Title/Abstract])) OR (neoplasm esophageal[Title/Abstract])) OR (esophagus neoplasm[Title/Abstract])) OR (esophagus neoplasms[Title/Abstract])) OR (neoplasm esophagus[Title/Abstract])) OR (neoplasms esophagus[Title/Abstract])) OR (neoplasms esophageal[Title/Abstract])) OR (cancer of esophagus[Title/Abstract])) OR (cancer of the esophagus[Title/Abstract])) OR (esophagus cancer[Title/Abstract])) OR (cancer esophagus[Title/Abstract])) OR (cancers esophagus[Title/Abstract])) OR (esophagus cancers[Title/Abstract])) OR (esophageal cancer[Title/Abstract])) OR (cancer esophageal[Title/Abstract])) OR (cancers esophageal[Title/Abstract])) OR (esophageal cancers[Title/Abstract])) AND ((((((((((((((((((((((((((((((((((Immune Checkpoint Inhibitors [MeSH Terms]) OR (checkpoint inhibitors immune [Title/Abstract])) OR (immune checkpoint inhibitor [Title/Abstract])) OR (checkpoint inhibitor immune[Title/Abstract])) OR (immune checkpoint blockers[Title/Abstract])) OR (checkpoint blockers immune[Title/Abstract])) OR (immune checkpoint blockade[Title/Abstract])) OR (checkpoint blockade immune[Title/Abstract])) OR (immune checkpoint inhibition[Title/Abstract))) OR (checkpoint inhibition immune[Title/Abstract])) OR (pd l1 inhibitors[Title/Abstract])) OR (pd l1 inhibitors[Title/Abstract])) OR (pd l1 inhibitor[Title/Abstract])) OR (pd l1 inhibitor[Title/Abstract])) OR (programmed death ligand 1 inhibitors[Title/Abstract])) OR (programmed death ligand 1 inhibitors[Title/Abstract])) OR (pd 1 pd l1 blockade[Title/Abstract])) OR (blockade pd 1 pd l1[Title/Abstract])) OR (pd 1 pd l1 blockade[Title/Abstract])) OR (ctla 4 inhibitors[Title/Abstract])) OR (ctla 4 inhibitors[Title/Abstract])) OR (ctla 4 inhibitor[Title/Abstract])) OR (ctla 4 inhibitor[Title/Abstract])) OR (cytotoxic t lymphocyte associated protein 4 inhibitors[Title/Abstract])) OR (cytotoxic t lymphocyte associated protein 4 inhibitors[Title/Abstract])) OR (cytotoxic t lymphocyte associated protein 4 inhibitor[Title/Abstract])) OR (cytotoxic t lymphocyte associated protein 4 inhibitor[Title/Abstract])) OR (pd 1 inhibitors[Title/Abstract])) OR (pd 1 inhibitors[Title/Abstract])) OR (pd 1 inhibitor[Title/Abstract])) OR (inhibitor pd 1[Title/Abstract])) OR (pd 1 inhibitor[Title/Abstract])) OR (programmed cell death protein 1 inhibitor[Title/Abstract])) OR (programmed cell death protein 1 inhibitors[Title/Abstract])) AND (((((((((randomized controlled trail[Title/Abstract]) OR (controlled clinical trail[Title/Abstract])) OR (trail[Title/Abstract])) OR (groups[Title/Abstract])) OR (phase[Title/Abstract])) OR (randomly[Title/Abstract])) OR (randomized[Title/Abstract])) OR (placebo[Title/Abstract])) OR (drug therapy[Title/Abstract])) |
| **Web of Science** | 1: TS= (Esophageal Neoplasms OR esophageal neoplasm OR neoplasm esophageal OR esophagus neoplasm OR esophagus neoplasms OR neoplasm esophagus OR neoplasms esophagus OR neoplasms esophageal OR cancer of esophagus OR cancer of the esophagus OR esophagus cancer OR cancer esophagus OR cancers esophagus OR esophagus cancers OR esophageal cancer OR cancer esophageal OR cancers esophageal OR esophageal cancers) and Preprint Citation Index (Exclude – Database) Results: 173269  2: TS=(Immune Checkpoint Inhibitors OR checkpoint inhibitors immune OR immune checkpoint inhibitor OR checkpoint inhibitor immune OR immune checkpoint blockers OR checkpoint blockers immune OR immune checkpoint blockade OR checkpoint blockade immune OR immune checkpoint inhibition OR checkpoint inhibition immune OR pd l1 inhibitors OR pd l1 inhibitors OR pd l1 inhibitor OR pd l1 inhibitor OR programmed death ligand 1 inhibitors OR programmed death ligand 1 inhibitors OR pd 1 pd l1 blockade OR blockade pd 1 pd l1 OR pd 1 pd l1 blockade OR ctla 4 inhibitors OR ctla 4 inhibitors OR ctla 4 inhibitor OR ctla 4 inhibitor OR cytotoxic t lymphocyte associated protein 4 inhibitors OR cytotoxic t lymphocyte associated protein 4 inhibitors OR cytotoxic t lymphocyte associated protein 4 inhibitor OR cytotoxic t lymphocyte associated protein 4 inhibitor OR pd 1 inhibitors OR pd 1 inhibitors OR pd 1 inhibitor OR inhibitor pd 1 OR pd 1 inhibitor OR programmed cell death protein 1 inhibitor OR programmed cell death protein 1 inhibitors) and Preprint Citation Index(Exclude – Database) Results: 131923  3: TS=(randomized controlled trail OR controlled clinical trail OR trail OR groups OR phase OR randomly OR randomized OR placebo OR drug therapy) and Preprint Citation Index (Exclude – Database) Results: 29024516  4: (((#1) AND #2) AND #3) and Preprint Citation Index (Exclude – Database) Results: 1392 |
| **Cochrane** | #1 MeSH descriptor: [Esophageal Neoplasms] explode all trees 2684  #2 (Neoplasm, Esophageal):ti,ab,kw (Word variations have been searched) 3552  #3 (Esophagus Neoplasm):ti,ab,kw (Word variations have been searched) 1990  #4 (Esophageal Neoplasm):ti,ab,kw (Word variations have been searched) 3552  #5 (Esophagus Neoplasms):ti,ab,kw (Word variations have been searched) 1990  #6 (Neoplasm, Esophagus):ti,ab,kw (Word variations have been searched) 1990  #7 (Neoplasms, Esophagus):ti,ab,kw (Word variations have been searched) 1990  #8 (Neoplasms, Esophageal):ti,ab,kw (Word variations have been searched) 3327  #9 (Cancer of Esophagus):ti,ab,kw (Word variations have been searched) 3247  #10 (Cancer of the Esophagus):ti,ab,kw (Word variations have been searched) 3378  #11 (Esophagus Cancer):ti,ab,kw (Word variations have been searched) 3378  #12 (Cancer, Esophagus):ti,ab,kw (Word variations have been searched) 3378  #13 (Cancers, Esophagus):ti,ab,kw (Word variations have been searched) 3378  #14 (Esophagus Cancers):ti,ab,kw (Word variations have been searched) 3378  #15 (Esophageal Cancer):ti,ab,kw (Word variations have been searched) 5801  #16 (Cancer, Esophageal):ti,ab,kw (Word variations have been searched) 5801  #17 (Cancers, Esophageal):ti,ab,kw (Word variations have been searched) 5801  #18 (Esophageal Cancers):ti,ab,kw (Word variations have been searched) 5801  #19 #1 or #2 or #3 or #4 or #5 or #6 or #7 or #8 or #9 or #10 or #11 or #12 or #13 or #14 or #15 or #16 or #17 or #18 7671  #20 MeSH descriptor: [Immune Checkpoint Inhibitors] explode all trees 322  #21 (Checkpoint Inhibitors, Immune):ti,ab,kw (Word variations have been searched) 2493  #22 (Immune Checkpoint Inhibitor):ti,ab,kw (Word variations have been searched) 2491  #23 (Checkpoint Inhibitor, Immune):ti,ab,kw (Word variations have been searched) 2491  #24 (Immune Checkpoint Blockers):ti,ab,kw (Word variations have been searched) 29  #25 (Checkpoint Blockers, Immune):ti,ab,kw (Word variations have been searched) 29  #26 (Immune Checkpoint Blockade):ti,ab,kw (Word variations have been searched) 542  #27 (Checkpoint Blockade, Immune):ti,ab,kw (Word variations have been searched) 542  #28 (Immune Checkpoint Inhibition):ti,ab,kw (Word variations have been searched) 688  #29 (Checkpoint Inhibition, Immune):ti,ab,kw (Word variations have been searched) 688  #30 (PD-L1 Inhibitors):ti,ab,kw (Word variations have been searched) 1776  #31 (PD L1 Inhibitors):ti,ab,kw (Word variations have been searched) 1836  #32 (PD L1 Inhibitor):ti,ab,kw (Word variations have been searched) 1835  #33 (PD-L1 Inhibitor):ti,ab,kw (Word variations have been searched) 1775  #34 (Programmed Death-Ligand 1 Inhibitors):ti,ab,kw (Word variations have been searched) 384  #35 (Programmed Death Ligand 1 Inhibitors):ti,ab,kw (Word variations have been searched) 547  #36 (PD 1 PD L1 Blockade):ti,ab,kw (Word variations have been searched) 430  #37 (CTLA-4 Inhibitors):ti,ab,kw (Word variations have been searched) 358  #38 (CTLA 4 Inhibitors):ti,ab,kw (Word variations have been searched) 361  #39 (CTLA-4 Inhibitor):ti,ab,kw (Word variations have been searched) 358  #40 (CTLA 4 Inhibitor or Cytotoxic T-Lymphocyte-Associated Protein 4 Inhibitors or Cytotoxic T Lymphocyte Associated Protein 4 Inhibitors or Cytotoxic T-Lymphocyte-Associated Protein 4 Inhibitor):ti,ab,kw (Word variations have been searched) 397  #41 (Cytotoxic T Lymphocyte Associated Protein 4 Inhibitor or PD-1 Inhibitors or PD 1 Inhibitors or PD-1 Inhibitor or Inhibitor, PD-1 or PD 1 Inhibitor or Programmed Cell Death Protein 1 Inhibitor orProgrammed Cell Death Protein 1 Inhibitors):ti,ab,kw (Word variations have been searched) 7241  #42 #20 or #21 or #22 or #23 or #24 or #25 or #26 or #27 or #28 or #29 or #30 or #31 or #32 or #33 or #34 or #35 or #36 or #37 or #38 or #39 or #40 or #41 9133  #49 #19 and #42 188 |
| **Embase** | #56. ('esophagus tumor'/exp OR 'esophageal 529 5 Dec 2024  neoplasm':ab,ti OR 'neoplasm, esophageal':ab,ti  OR 'esophagus neoplasm':ab,ti OR 'esophagus  neoplasms':ab,ti OR 'neoplasm, esophagus':ab,ti  OR 'neoplasms, esophagus':ab,ti OR 'neoplasms,  esophageal':ab,ti OR 'cancer of esophagus':ab,ti  OR 'cancer of the esophagus':ab,ti OR 'esophagus  cancer':ab,ti OR 'cancer, esophagus':ab,ti OR  'cancers, esophagus':ab,ti OR 'esophagus  cancers':ab,ti OR 'esophageal cancer':ab,ti OR  'cancer, esophageal':ab,ti OR 'cancers,  esophageal':ab,ti OR 'esophageal cancers':ab,ti)  AND ('immune checkpoint inhibitor' OR 'checkpoint  inhibitors, immune':ab,ti OR 'immune checkpoint  inhibitor':ab,ti OR 'checkpoint inhibitor,  immune':ab,ti OR 'immune checkpoint  blockers':ab,ti OR 'checkpoint blockers,  immune':ab,ti OR 'immune checkpoint  blockade':ab,ti OR 'checkpoint blockade,  immune':ab,ti OR 'checkpoint blockade,  immune':ab,ti OR 'checkpoint blockade,  immune':ab,ti OR 'pd-l1 inhibitors':ab,ti OR 'pd  l1 inhibitors':ab,ti OR 'pd-l1 inhibitor':ab,ti  OR 'pd l1 inhibitor':ab,ti OR 'programmed  death-ligand 1 inhibitors':ab,ti OR 'programmed  death ligand 1 inhibitors':ab,ti OR 'pd-1-pd-l1  blockade':ab,ti OR 'blockade, pd-1-pd-l1':ab,ti  OR 'pd 1 pd l1 blockade':ab,ti OR 'ctla-4  inhibitors':ab,ti OR 'ctla 4 inhibitors':ab,ti OR  'ctla-4 inhibitor':ab,ti OR 'ctla-4  inhibitor':ab,ti OR 'cytotoxic  t-lymphocyte-associated protein 4  inhibitors':ab,ti OR 'cytotoxic t lymphocyte  associated protein 4 inhibitors':ab,ti OR  'cytotoxic t-lymphocyte-associated protein 4  inhibitor':ab,ti OR 'cytotoxic t lymphocyte  associated protein 4 inhibitor':ab,ti OR 'pd-1  inhibitors':ab,ti OR 'pd 1 inhibitors':ab,ti OR  'pd-1 inhibitor':ab,ti OR 'inhibitor, pd-1':ab,ti  OR 'inhibitor, pd-1':ab,ti OR 'programmed cell  death protein 1 inhibitor':ab,ti OR 'programmed  cell death protein 1 inhibitors':ab,ti) AND  ('randomized controlled trail':ab,ti OR  'controlled clinical trail':ab,ti OR  'trail':ab,ti OR 'groups':ab,ti OR 'phase':ab,ti  OR 'randomly':ab,ti OR 'randomized':ab,ti OR  'placebo':ab,ti OR 'drug therapy':ab,ti)  #55. 'randomized controlled trail':ab,ti OR 6,446,693 5 Dec 2024  'controlled clinical trail':ab,ti OR  'trail':ab,ti OR 'groups':ab,ti OR 'phase':ab,ti  OR 'randomly':ab,ti OR 'randomized':ab,ti OR  'placebo':ab,ti OR 'drug therapy':ab,ti  #54. 'immune checkpoint inhibitor' OR 'checkpoint 61,733 5 Dec 2024  inhibitors, immune':ab,ti OR 'immune checkpoint  inhibitor':ab,ti OR 'checkpoint inhibitor,  immune':ab,ti OR 'immune checkpoint  blockers':ab,ti OR 'checkpoint blockers,  immune':ab,ti OR 'immune checkpoint  blockade':ab,ti OR 'checkpoint blockade,  immune':ab,ti OR 'checkpoint blockade,  immune':ab,ti OR 'checkpoint blockade,  immune':ab,ti OR 'pd-l1 inhibitors':ab,ti OR 'pd  l1 inhibitors':ab,ti OR 'pd-l1 inhibitor':ab,ti  OR 'pd l1 inhibitor':ab,ti OR 'programmed  death-ligand 1 inhibitors':ab,ti OR 'programmed  death ligand 1 inhibitors':ab,ti OR 'pd-1-pd-l1  blockade':ab,ti OR 'blockade, pd-1-pd-l1':ab,ti  OR 'pd 1 pd l1 blockade':ab,ti OR 'ctla-4  inhibitors':ab,ti OR 'ctla 4 inhibitors':ab,ti OR  'ctla-4 inhibitor':ab,ti OR 'ctla-4  inhibitor':ab,ti OR 'cytotoxic  t-lymphocyte-associated protein 4  inhibitors':ab,ti OR 'cytotoxic t lymphocyte  associated protein 4 inhibitors':ab,ti OR  'cytotoxic t-lymphocyte-associated protein 4  inhibitor':ab,ti OR 'cytotoxic t lymphocyte  associated protein 4 inhibitor':ab,ti OR 'pd-1  inhibitors':ab,ti OR 'pd 1 inhibitors':ab,ti OR  'pd-1 inhibitor':ab,ti OR 'inhibitor, pd-1':ab,ti  OR 'inhibitor, pd-1':ab,ti OR 'programmed cell  death protein 1 inhibitor':ab,ti OR 'programmed  cell death protein 1 inhibitors':ab,ti  #53. 'programmed cell death protein 1 82 5 Dec 2024  inhibitors':ab,ti  #52. 'programmed cell death protein 1 inhibitor':ab,ti 119 5 Dec 2024  #51. 'inhibitor, pd-1':ab,ti 79 5 Dec 2024  #50. 'inhibitor, pd-1':ab,ti 79 5 Dec 2024  #49. 'pd-1 inhibitor':ab,ti 3,757 5 Dec 2024  #48. 'pd 1 inhibitors':ab,ti 3,202 5 Dec 2024  #47. 'pd-1 inhibitors':ab,ti 3,203 5 Dec 2024  #46. 'cytotoxic t lymphocyte associated protein 4 20 5 Dec 2024  inhibitor':ab,ti  #45. 'cytotoxic t-lymphocyte-associated protein 4 20 5 Dec 2024  inhibitor':ab,ti  #44. 'cytotoxic t lymphocyte associated protein 4 22 5 Dec 2024  inhibitors':ab,ti  #43. 'cytotoxic t-lymphocyte-associated protein 4 22 5 Dec 2024  inhibitors':ab,ti  #42. 'ctla-4 inhibitor':ab,ti 405 5 Dec 2024  #41. 'ctla-4 inhibitor':ab,ti 405 5 Dec 2024  #40. 'ctla 4 inhibitors':ab,ti 530 5 Dec 2024  #39. 'ctla-4 inhibitors':ab,ti 531 5 Dec 2024  #38. 'pd 1 pd l1 blockade':ab,ti 1,140 5 Dec 2024  #37. 'blockade, pd-1-pd-l1':ab,ti 5 5 Dec 2024  #36. 'pd-1-pd-l1 blockade':ab,ti 1,140 5 Dec 2024  #35. 'programmed death ligand 1 inhibitors':ab,ti 104 5 Dec 2024  #34. 'programmed death-ligand 1 inhibitors':ab,ti 106 5 Dec 2024  #33. 'pd l1 inhibitor':ab,ti 1,867 5 Dec 2024  #32. 'pd-l1 inhibitor':ab,ti 1,867 5 Dec 2024  #31. 'pd l1 inhibitors':ab,ti 3,014 5 Dec 2024  #30. 'pd-l1 inhibitors':ab,ti 3,015 5 Dec 2024  #29. 'checkpoint blockade, immune':ab,ti 12 5 Dec 2024  #28. 'checkpoint blockade, immune':ab,ti 12 5 Dec 2024  #27. 'checkpoint blockade, immune':ab,ti 12 5 Dec 2024  #26. 'immune checkpoint blockade':ab,ti 11,227 5 Dec 2024  #25. 'checkpoint blockers, immune':ab,ti 16 5 Dec 2024  #24. 'immune checkpoint blockers':ab,ti 741 5 Dec 2024  #23. 'checkpoint inhibitor, immune':ab,ti 16 5 Dec 2024  #22. 'immune checkpoint inhibitor':ab,ti 13,429 5 Dec 2024  #21. 'checkpoint inhibitors, immune':ab,ti 54 5 Dec 2024  #20. 'immune checkpoint inhibitor' 37,520 5 Dec 2024  #19. 'esophagus tumor'/exp OR 'esophageal 121,819 5 Dec 2024  neoplasm':ab,ti OR 'neoplasm, esophageal':ab,ti  OR 'esophagus neoplasm':ab,ti OR 'esophagus  neoplasms':ab,ti OR 'neoplasm, esophagus':ab,ti  OR 'neoplasms, esophagus':ab,ti OR 'neoplasms,  esophageal':ab,ti OR 'cancer of esophagus':ab,ti  OR 'cancer of the esophagus':ab,ti OR 'esophagus  cancer':ab,ti OR 'cancer, esophagus':ab,ti OR  'cancers, esophagus':ab,ti OR 'esophagus  cancers':ab,ti OR 'esophageal cancer':ab,ti OR  'cancer, esophageal':ab,ti OR 'cancers,  esophageal':ab,ti OR 'esophageal cancers':ab,ti  #18. 'esophageal cancers':ab,ti 2,766 5 Dec 2024  #17. 'cancers, esophageal':ab,ti 101 5 Dec 2024  #16. 'cancer, esophageal':ab,ti 803 5 Dec 2024  #15. 'esophageal cancer':ab,ti 38,002 5 Dec 2024  #14. 'esophagus cancers':ab,ti 88 5 Dec 2024  #13. 'cancers, esophagus':ab,ti 38 5 Dec 2024  #12. 'cancer, esophagus':ab,ti 126 5 Dec 2024  #11. 'esophagus cancer':ab,ti 874 5 Dec 2024  #10. 'cancer of the esophagus':ab,ti 2,010 5 Dec 2024  #9. 'cancer of esophagus':ab,ti 147 5 Dec 2024  #8. 'neoplasms, esophageal':ab,ti 20 5 Dec 2024  #7. 'neoplasms, esophagus':ab,ti 4 5 Dec 2024  #6. 'neoplasm, esophagus':ab,ti 5 5 Dec 2024  #5. 'esophagus neoplasms':ab,ti 11 5 Dec 2024  #4. 'esophagus neoplasm':ab,ti 4 5 Dec 2024  #3. 'neoplasm, esophageal':ab,ti 11 5 Dec 2024  #2. 'esophageal neoplasm':ab,ti 232 5 Dec 2024  #1. 'esophagus tumor'/exp 118,657 5 Dec 2024 |

Table S3. Results of Head-to-head Meta-analysis According to Pairwise Meta-analysis and Comparisons Between bayesian Network Meta-analysis

| **Table S3. Results of Head-to-head Meta-analysis According to Pairwise Meta-analysis and Comparisons Between bayesian Network Meta-analysis** | | | | | | |
| --- | --- | --- | --- | --- | --- | --- |
| **Subgroup analysis  of overall survival** | **PD-L1 all** | | **PD-L1 ≥ 10%** | | **PD-L1 < 10%** | |
|  | **HR for PWNA** | **HR for NMA** | **HR for PWNA** | **HR for NMA** | **HR for PWNA** | **HR for NMA** |
| Sintilimab | 0.70(0.50,0.97) | 0.70(0.50,0.97) | 0.74(0.40,1.42) | 0.74(0.39,1.39) | 0.95(0.56,1.58) | 0.95(0.57,1.60) |
| Tislelizumab | 0.70(0.57,0.85) | 0.70(0.57,0.85) | 0.53(0.37,0.77) | 0.53(0.37,0.76) | 0.85(0.65,1.11) | 0.85(0.65,1.11) |
| Pembrolizumab | 0.77(0.63.0.96) | 0.77(0.62,0.96) | 0.64(0.46,0.90) | 0.64(0.46,0.90) | 0.88(0.66,1.16) | 0.88(0.66,1.17) |
| Nivolumab | 0.77(0.62,0.96) | 0.77(0.62,0.96) | 0.69(0.46,1.04) | 0.69(0.46,1.04) | 0.80(0.62-1.04) | 0.80(0.62,1.04) |
| Camrelizumab | 0.71(0.57,0.87) | 0.71(0.57,0.88) | 0.60(0.32,1.08) | 0.60(0.33,1.10) | 0.77(0.57,0.89) | 0.71(0.57,0.89) |

Table S4. baseline Clinical and disease Characteristics of Trials Included in the Network Meta-analysis

| **Table S4. baseline Clinical and disease Characteristics of Trials Included in the Network Meta-analysis** | | | | | | | | | | | |
| --- | --- | --- | --- | --- | --- | --- | --- | --- | --- | --- | --- |
|  | **ORIENT-2** | | **RATIONALE-302** | | **KEYNOTE-181** | | **ATTRACTION-3** | | **ESCORT** | | |
|  | **Intervention Arm** | **Control Arm** | **Intervention Arm** | **Control Arm** | **Intervention Arm** | **Control Arm** | **Intervention Arm** | **Control Arm** | **Intervention Arm** | **Control Arm** |  |
| **PD-L1 expression level (CPS)** | | | | | | | | | | | |
| <1 | 11.6% | 17.9% | / | / | / | / | 52.0% | 51.0% | / | / |  |
| ≥1 | 66.3% | 52.6% | / | / | / | / | 48.0% | 49.0% | / | / |  |
| <10 | 35.8% | 49.5% | / | / | 64.0% | 62.4% | 70.0% | 73.0% | / | / |  |
| ≥10 | 42.1% | 21.1% | / | / | 34.1% | 36.6% | 30.0% | 27.0% | / | / |  |
| **PD-L1 expression level (TPS)** | | | | | | | | | | | |
| <1% | 46.3% | 45.3% | / | / | / | / | / | / | 57.0% | 54.0% |  |
| ≥1% | 31.6% | 25.3% | / | / | / | / | / | / | 41.0% | 45.0% |  |
| <10% | 63.2% | 55.8% | 45.3% | 54.7% | / | / | / | / | 86.0% | 82.0% |  |
| ≥10% | 14.7% | 14.7% | 34.8% | 26.6% | / | / | / | / | 11.0% | 16.0% |  |
| **Gender** | | | | | | | | | | | |
| Male | 92.6% | 88.4% | 84.8% | 84.0% | 86.9% | 86.3% | 85.0% | 89.0% | 91.0% | 87.0% |  |
| Female | 7.4% | 11.6% | 15.2% | 16.0% | 13.1% | 13.7% | 15.0% | 11.0% | 9.0% | 13.0% |  |
| **ECOG** | | | | | | | | | | | |
| 0 | 24.2% | 24.2% | 25.8% | 23.4% | 40.1% | 36.9% | 48.0% | 51.0% | 20.0% | 20.0% |  |
| 1 | 75.8% | 75.8% | 74.2% | 76.6% | 59.6% | 62.7% | 52.0% | 49.0% | 80.0% | 80.0% |  |
| **Previous treatment** | | | | | | | | | | | |
| First-line chemotherapy | 88.4% | 89.5% | / | / | / | / | / | / | 96.0% | 93.0% |  |
| Radiotherapy | 52.6% | 61.1% | 66.0% | 63.7% | / | / | 73.0% | 68.0% | 68.0% | 63.0% |  |
| Surgery | 61.1% | 49.5% | 36.7% | 38.7% | / | / | 53.0% | 45.0% | 50.0% | 48.0% |  |
| **Site of metastases** | | | | | | | | | | | |
| Lymph node | 70.5% | 67.4% | / | / | / | / | 76.0% | 78.0% | 83.0% | 88.0% |  |
| Liver | 28.4% | 34.7% | / | / | / | / | 27.0% | 26.0% | 24.0% | 21.0% |  |
| Lung | 38.9% | 48.4% | / | / | / | / | 47.0% | 44.0% | 47.0% | 43.0% |  |
| Bone | 6.3% | 6.3% | / | / | / | / | 11.0% | 12.0% | 13.0% | 13.0% |  |
| Other | 32.6% | 33.7% | / | / | / | / | / | / | 20.0% | 17.0% |  |

Table S5. Incidence of Treatment-related Adverse Events in Each Immunotherapy Regimen

| **Table S5. Incidence of Treatment-related Adverse Events in Each Immunotherapy Regimen** | | | | | | | | | | | | | | |
| --- | --- | --- | --- | --- | --- | --- | --- | --- | --- | --- | --- | --- | --- | --- |
| **Treatment-related AEs** | Anemia | WBC count decreased | Neutrophils count decreased | Nausea | Decreased appetite | Diarrhea | Vomiting | Hypothyroidism | Fatigue | Pulmonary inflammation | Lung infection | AST increased | Abnormal liver function | interstitial lung disease |
| Sintilimab | 8.50% | 8.50% | 5.30% | 3.20% | 1.10% | 4.30% | 2.10% | 12.80% | 5.30% | 10.60% | 2.10% | 7.40% | 6.40% |  |
| Tislelizumab | 11% | 2% | 1.20% | 2.70% | 6.30% | 5.50% | 1.60% | 10.20% | 7.50% |  |  | 11.40% |  |  |
| Nivolumab | 3% | 2% | 2% | 2% | 8% | 11% |  |  | 8% |  |  |  |  | 2% |
| Camrelizumab | 11% | 8% | 4% | 2% | 5% | 5% | 1% | 17% |  |  | 1% |  |  |  |

Table S6. Ranking Profiles in the Bayesian Network Meta-analysis (Overall Survival)

| **Table S6. Ranking Profiles in the Bayesian Network Meta-analysis (Overall Survival)** | | | | | | |
| --- | --- | --- | --- | --- | --- | --- |
| **Overall Survival** | **Rank probability (%)** | | | | | |
|  | **Rank1^st^** | **Rank2^nd^** | **Rank3^rd^** | **Rank4^th^** | **Rank5^th^** | **Rank6^th^** |
| **Sintilimab** | 35.19% | 18.04% | 15.03% | 14.18% | 15.83% | 1.72% |
| **Tislelizumab** | 26.72% | 28.40% | 22.16% | 14.78% | 7.91% | 0.02% |
| **Pembrolizumab** | 7.26% | 13.65% | 20.17% | 27.39% | 30.79% | 7.46% |
| **Nivolumab** | 7.90% | 13.70% | 19.65% | 26.53% | 31.30% | 0.92% |
| **Camrelizumab** | 22.93% | 26.20% | 22.99% | 17.08% | 10.72% | 0.07% |
| **Chemotherapy** | 0.00% | 0.00% | 0.00% | 0.03% | 3.45% | 96.51% |

Table S7. Ranking Profiles in the Bayesian Network Meta-analysis (Progression-free Survival)

| **Table S7. Ranking Profiles in the Bayesian Network Meta-analysis (Progression-free Survival)** | | | | | | |
| --- | --- | --- | --- | --- | --- | --- |
| **Progression-free Survival** | **Rank probability (%)** | | | | | |
|  | Rank1^st^ | Rank2^nd^ | Rank3^rd^ | Rank4^th^ | Rank5^th^ | Rank6^th^ |
| **Sintilimab** | 2.68% | 19.46% | 18.86% | 15.37% | 19.34% | 24.28% |
| **Tislelizumab** | 7.29% | 25.66% | 16.35% | 11.76% | 14.67% | 24.26% |
| **Pembrolizumab** | 2.19% | 36.11% | 31.53% | 16.23% | 9.56% | 4.38% |
| **Nivolumab** | 0.10% | 4.42% | 9.65% | 15.28% | 28.90% | 41.65% |
| **Camrelizumab** | 87.74% | 10.76% | 1.30% | 0.18% | 0.02% | 0.00% |
| **Chemotherapy** | 0.00% | 3.57% | 22.31% | 41.17% | 27.52% | 5.42% |

Table S8. Ranking Profiles in the Bayesian Network Meta-analysis (Objective Response Rate)

| **Table S8. Ranking Profiles in the Bayesian Network Meta-analysis (Objective Response Rate)** | | | | | |
| --- | --- | --- | --- | --- | --- |
| **Objective Response Rate** | **Rank probability (%)** | | | | |
|  | **Rank1^st^** | **Rank2^nd^** | **Rank3^rd^** | **Rank4^th^** | **Rank5^th^** |
| **Sintilimab** | 29.80% | 23.90% | 37.50% | 4.80% | 3.90% |
| **Tislelizumab** | 29.80% | 41.00% | 29.00% | 0.30% | 0.00% |
| **Pembrolizumab** | 40.40% | 34.70% | 23.90% | 0.70% | 0.20% |
| **Nivolumab** | 0.00% | 0.30% | 4.50% | 28.40% | 66.80% |
| **Chemotherapy** | 0.00% | 0.00% | 5.10% | 65.80% | 29.10% |

Table S9. Ranking Profiles in the Bayesian Network Meta-analysis (Adverse Events)

| **Table S9. Ranking Profiles in the Bayesian Network Meta-analysis (Adverse Events)** | | | | | |
| --- | --- | --- | --- | --- | --- |
| **Adverse Events** | **Rank probability (%)** | | | | |
|  | **Rank1^st^** | **Rank2^nd^** | **Rank3^rd^** | **Rank4^th^** | **Rank5^th^** |
| **Sintilimab** | 0.00% | 0.50% | 43.00% | 34.60% | 21.90% |
| **Tislelizumab** | 8.30% | 91.30% | 0.50% | 0.00% | 0.00% |
| **Nivolumab** | 91.70% | 8.30% | 0.00% | 0.00% | 0.00% |
| **Camrelizumab** | 0.00% | 0.00% | 3.00% | 21.20% | 75.80% |
| **Chemotherapy** | 0.00% | 0.00% | 53.50% | 44.20% | 2.20% |

Table S10. Ranking Profiles in the Bayesian Network Meta-analysis (grade ≥ 3 AEs)

| **Table S10. Ranking Profiles in the Bayesian Network Meta-analysis (grade ≥ 3 AEs)** | | | | | |
| --- | --- | --- | --- | --- | --- |
| **grade ≥ 3 AEs** | **Rank probability (%)** | | | | |
|  | **Rank1^st^** | **Rank2^nd^** | **Rank3^rd^** | **Rank4^th^** | **Rank5^th^** |
| **Sintilimab** | 0.00% | 0.00% | 0.00% | 14.10% | 85.90% |
| **Tislelizumab** | 10.70% | 88.30% | 0.90% | 0.00% | 0.00% |
| **Nivolumab** | 89.30% | 10.60% | 0.10% | 0.00% | 0.00% |
| **Camrelizumab** | 0.00% | 1.00% | 98.90% | 0.00% | 0.00% |
| **Chemotherapy** | 0.00% | 0.00% | 0.00% | 85.90% | 14.10% |


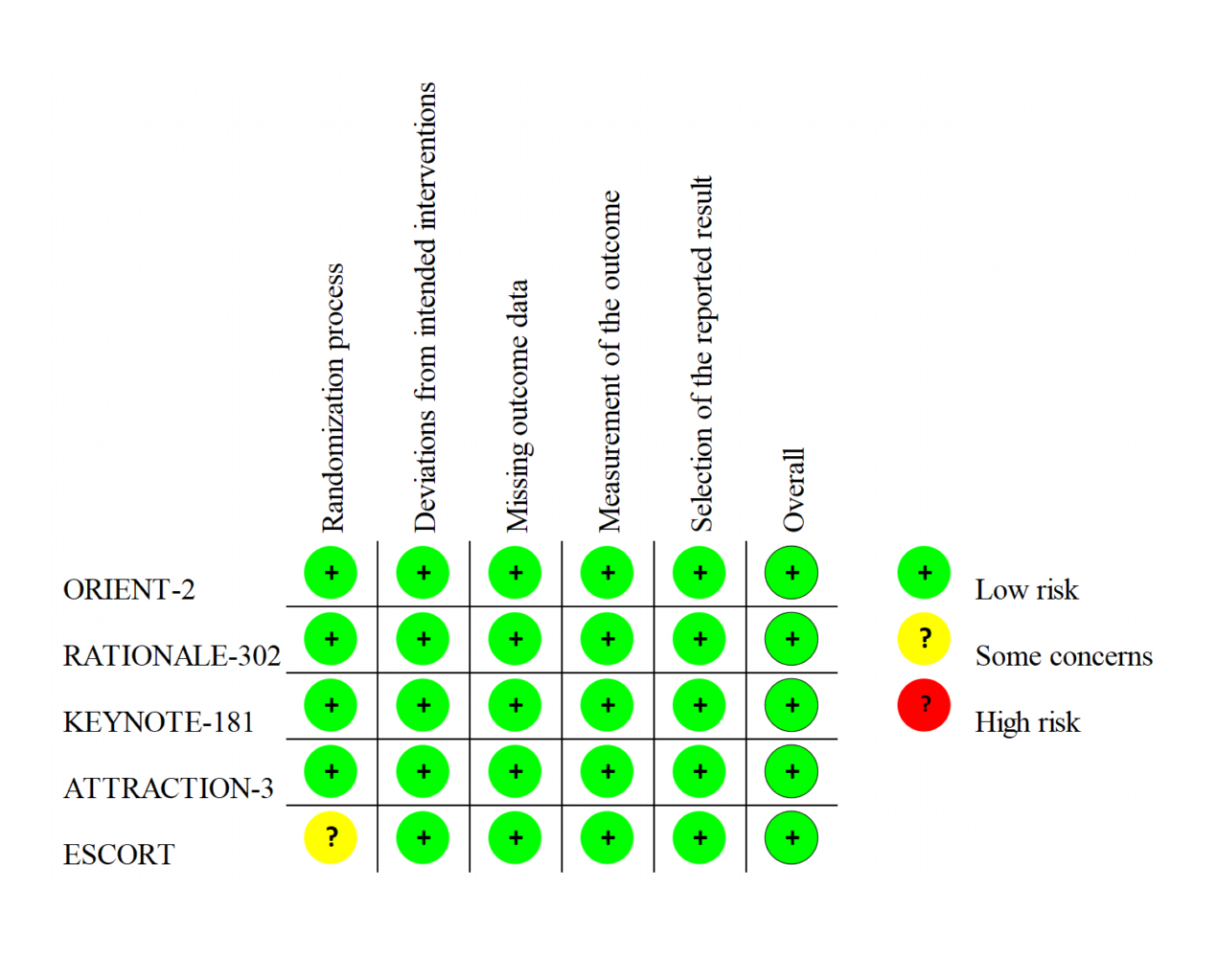


**Figure S1. Summary of results from assessment of studies by using Cochrane Risk of Bias Tool 2.0.**Studies were classified into one of three categories: low, high risk or having “some concerns”


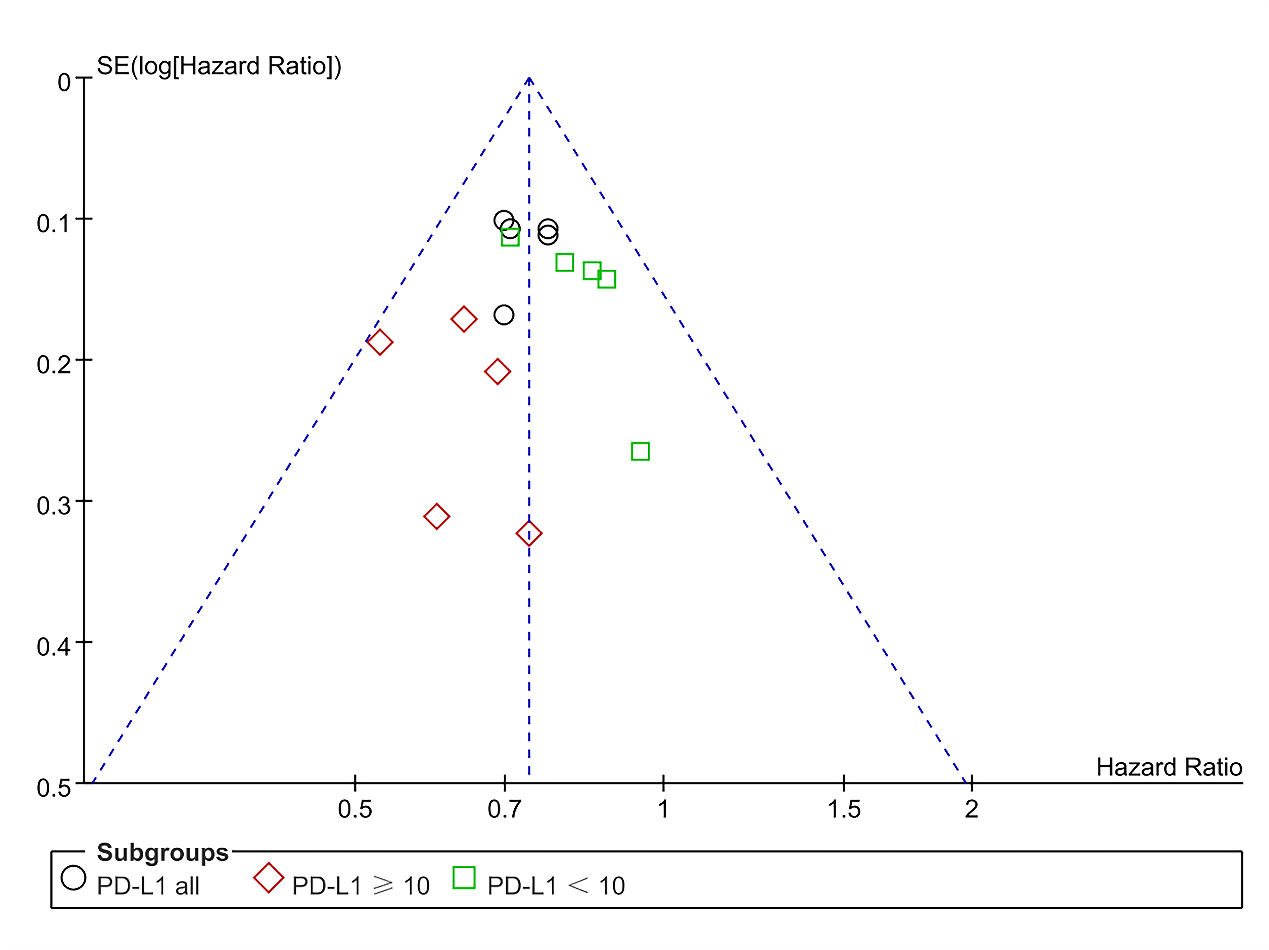


**Figure S2. Funnel plot to detect the publication bias of included studies**
